# Atypical maturation of the functional connectome hierarchy in autism

**DOI:** 10.1186/s13229-025-00641-9

**Published:** 2025-03-26

**Authors:** Jong-eun Lee, Sunghun Kim, Shinwon Park, Hyoungshin Choi, Bo-yong Park, Hyunjin Park

**Affiliations:** 1 Department of Electrical and Computer Engineering, Sungkyunkwan University, Suwon, Republic of Korea; 2 Center for Neuroscience Imaging Research, Institute for Basic Science, Suwon, Republic of Korea; 3 Autism Center, Child Mind Institute, New York, NY USA; 4 Department of Brain and Cognitive Engineering, Korea University, Seoul, Republic of Korea

**Keywords:** Autism spectrum disorder, Cortical hierarchy, Normative modeling, Integration and segregation

## Abstract

**Background:**

Autism spectrum disorder (ASD) is marked by disruptions in low-level sensory processing and higher-order sociocognitive functions, suggesting a complex interplay between different brain regions across the cortical hierarchy. However, the developmental trajectory of this hierarchical organization in ASD remains underexplored. Herein, we investigated the maturational abnormalities in the cortical hierarchy among individuals with ASD.

**Methods:**

Resting-state functional magnetic resonance imaging data from three large-scale datasets were analyzed: Autism Brain Imaging Data Exchange I and II and Lifespan Human Connectome Project Development (aged 5–22 years). The principal functional connectivity gradient representing cortical hierarchy was estimated using diffusion map embedding. By applying normative modeling with the generalized additive model for location, scale, and shape (GAMLSS), we captured the nonlinear trajectories of the developing functional gradient, as well as the individual-level deviations in ASD from typical development based on centile scores measured as deviations from the normative curves. A whole-brain summary metric, the functional hierarchy score, was derived to measure the extent of abnormal maturation in individuals with ASD. Finally, through a series of mediation analyses, we examined the potential role of network-level connectomic disruptions between the diagnoses and deviations in the cortical hierarchy.

**Results:**

The maturation of cortical hierarchy in individuals with ASD followed a non-linear trajectory, showing delayed maturation during childhood compared to that of typically developing individuals, followed by an accelerated “catch-up” phase during adolescence and a subsequent decline in young adulthood. The nature of these deviations varied across networks, with sensory and attention networks displaying the most pronounced abnormalities in childhood, while higher-order networks, particularly the default mode network (DMN), remaining impaired from childhood to adolescence. Mediation analyses revealed that the persistent reduction in DMN segregation throughout development was a key contributor to the atypical development of cortical hierarchy in ASD.

**Limitations:**

The uneven distribution of samples across age groups, particularly in the later stages of development, limited our ability to fully capture developmental trajectories among older individuals.

**Conclusions:**

These findings highlight the importance of understanding the developmental trajectories of cortical organization in ASD, collectively suggesting that early interventions aimed at promoting the normative development of higher-order networks may be critical for improving outcomes in individuals with ASD.

**Supplementary Information:**

The online version contains supplementary material available at 10.1186/s13229-025-00641-9.

## Background

Autism spectrum disorder (ASD) is a neurodevelopmental condition characterized by a wide range of behavioral phenotypes, including persistent deficits in social communication and interaction, repetitive behaviors, and restricted interests [1]. Over the past several decades, convergent evidence of impairment in social information processing has indicated that ASD is a socio-cognitive condition [2]. Recent studies have demonstrated that sensory processing may be central to understanding the neurobiology of ASD [3]. As such, a remaining challenge in ASD research is to identify the common neurobiological basis underlying its system-wide effects.

A range of approaches has been employed to comprehensively understand the complex behavioral phenotypes of ASD [4–6]. Recently, hierarchy-based measures have gained significant traction because of their promising findings and applicability [7, 8]. Central to this approach is the idea that the brain is systematically organized in a hierarchical manner, linking low-level sensory processing to higher-order cognitive functions [9]. Prior studies have consistently supported the idea that the brain’s hierarchical structure facilitates the transmission of sensory inputs across multiple cortical layers into transmodal regions, such as the default mode network (DMN) and frontoparietal network [10, 11]. Given that ASD manifests as a heterogeneous combination of perceptual and social symptoms, this hierarchy-based approach offers a comprehensive framework for unraveling its complexity. Notably, prior conceptualizations of ASD as a condition of altered long-range connectivity linking sensory-driven posterior regions to more integrative anterior ones have hinted at underlying hierarchical disorganization [12]. Additionally, other work has revealed that abnormal sensory-transmodal integration patterns correlate with clinical severity in ASD [13]. Recent studies using manifold learning in resting-state functional MRI (rs-fMRI) have further demonstrated that a principal eigenvector (i.e., gradient) of functional connectivity spans a continuous brain axis, with sensory and motor networks at one end and the DMN at the other end [9]. This principal gradient closely aligns with the key characteristics of ASD, including atypical social and communication abilities, repetitive behaviors and interests, and altered sensory and perceptual processing [7], making it a critical feature for understanding the underlying connectopathy of ASD. Indeed, a previous case-control study reported the global suppression of the connectome gradient in individuals with ASD, showing a narrower distribution of gradient values compared to controls, leading to a disturbed cortical hierarchy [7].

It has been well established that the whole-brain organization of the functional connectome is not static, but instead undergoes significant changes across the lifespan, particularly during childhood and adolescence [14, 15]. A previous study using longitudinal data showed that the maturation of the principal connectome gradient from childhood to adolescence entails both segregation and integration across various networks, and is associated with the expression of genes involved in calcium ion-regulated exocytosis and chemical synaptic transmission [16]. Despite relatively well-established developmental effects on the cortical gradient, the trajectory of atypical functional connectome organization in ASD remains understudied. Most existing studies have considered demographic factors (e.g., age and sex) as confounding variables during statistical analyses, neglecting the opportunity to directly assess developmental effects in the autistic brain. To fully capture the developmental dynamics underlying atypical functional connectome organization in ASD, it is crucial to move beyond static snapshots and adopt approaches that account for changes over developmental time. Trajectory-based frameworks offer a powerful means of exploring how deviations from typical developmental patterns unfold, providing critical insights into the hierarchical disruptions observed in ASD. Thus, our work focused on characterizing age-dependent changes in the principal connectome gradient in individuals with ASD, using age as the main variable of interest.

Trajectory analysis, which quantifies changes in brain structure or function with respect to age, is essential for understanding developmental phenomena in both typically developing (TD) brains, and those with psychiatric disorders [17, 18]. Given that typical brain development has been well characterized, trajectory analysis explains pathological disturbances as deviations from the typical developmental path. Specifically, deviant developmental trajectories are characterized by two distinct features: the timing and shape of the trajectory [18]. For example, one longitudinal neuroimaging study demonstrated the presence of early brain overgrowth (i.e., altered timing) in young children with a high familial risk of autism [19]. Alterations in the shape of a trajectory (e.g., halting, failure to mature, or ectopic development) may indicate more severe developmental disturbances [20]. While longitudinal data are considered the gold standard for mapping developmental trajectories, challenges, such as data collection difficulties and attrition, have led to the rise of a complementary approach: normative modeling. This method, which uses large-scale cross-sectional datasets across a wide age range, offers a promising tool for identifying individual-level abnormalities by measuring centile deviations from normative curves [21, 22]. These centile deviations represent the relative distance from the median of the age-specific distributions, with more extreme centile scores indicating a greater deviation from the norm. Notably, one recent study using normative modeling to whole-brain cortical thickness identified highly individualized patterns of abnormalities that would have been statistically overlooked using traditional case-control approaches [23].

In this study, we aimed to evaluate whether a distinct developmental trajectory reflecting an atypical cortical hierarchy exists in ASD compared with the TD group, primarily hypothesizing that the principal gradient would follow a maturational process. To investigate this, we first applied a normative modeling approach to the principal gradient of functional connectivity to trace the development of cortical hierarchy in TD individuals. In particular, a generalized additive model for location, scale, and shape (GAMLSS) [24] was used to establish a stable and flexible model capable of capturing nonlinear trajectories. Subsequently, we explored whether a summary metric, the hierarchy score, based on the trajectory of the whole-brain hierarchy in ASD relative to the normative model, would show a distinct pattern compared with the TD group. Finally, we performed mediation analyses to examine whether the degree of network integration/segregation mediated the relationship between diagnosis and hierarchy scores to gain connectome-level insights into how topological characteristics influence atypical developmental trajectories in ASD. Because our analyses are based on cross-sectional data, we use terms like “maturational abnormalities” and “developmental trajectories” to describe observed age-related associations at the population level, rather than definitive longitudinal changes in individual participants.

## Methods

### Study participants

Two large-scale independent datasets from the Autism Brain Imaging Data Exchange (ABIDE), termed the ABIDE I and II (http://fcon_1000.projects.nitrc.org/indi/abide) [25, 26], were analyzed. The ABIDE database provides a large number of samples obtained from multiple centers. Participants with large head motion in the rs-fMRI time series (≥ 0.5 mm mean framewise displacement) and faulty cortical surface segmentation in T1-weighted (T1w) MRI were excluded (Supplementary Fig. 1). ABIDE data collection was performed according to local Institutional Review Board guidelines. All ABIDE datasets were anonymized, and no protected health information was included, following the Health Insurance Portability and Accountability Act guidelines and the 1000 Functional Connectomes Project or International Neuroimaging Data-sharing Initiative protocols. The site-specific details of demographic information are described in Supplementary Tables 1–3.

To construct a normative developmental trajectory, we used an additional cohort of 652 neurologically healthy children and adolescents from the Lifespan Human Connectome Project Development (HCP-D) dataset [27]. These participants (aged 5–21 years [14.44 ± 4.06, mean ± standard deviation], 301 males and 251 females) represent TD individuals with diverse ethnic, racial, and socioeconomic backgrounds. The HCP-D data collection was approved by the local Institutional Review Board. All participants aged 18 and above provided written informed consent to participate, while for children under 18 years of age, a parent or legal guardian provided written informed consent to participate.

### Data acquisition

Both T1w structural MRI and rs-fMRI were available from all sites in both the discovery (ABIDE-I) and replication (ABIDE-II) datasets. The site-specific imaging acquisition parameters are presented in Supplementary Tables 4 and 5. For the HCPD study, brain imaging data were collected using a 3T Siemens Prisma scanner (Siemens, Erlangen, Germany). T1w and T2-weighted (T2w) images were acquired using multi-echo magnetization prepared rapid acquisition gradient echo (MPRAGE) at 0.8 mm isotropic (T1w: repetition time [TR] = 2,500 ms; echo time [TE] = 1.81 ms; inversion time [TI] = 1,000 ms; flip angle = 8°; T2w: TR = 3,200 ms; TE = 564 ms). Rs-fMRI data were obtained with the sequences counterbalanced using anterior-posterior and posterior-anterior phase-encoding directions at 2 mm isotropic, with a multi-band factor of 8 (TR = 800 ms; TE = 37 ms; flip angle = 52°; 488 volumes).

### Data preprocessing

In the HCP-D dataset, T1w structural MRI data were bias-field-corrected and skull stripped, following which the cortical surfaces were reconstructed. Structural images were segmented into the cerebrospinal fluid, gray matter, and white matter, and then spatially normalized to the standard Montreal Neurological Institute (MNI) space. Blood oxygen level-dependent (BOLD) time-series data underwent slice-timing correction, rigid-body transformation-based motion correction, intensity inhomogeneity correction, co-registration with structural images, normalization to the MNI space, and projection onto the cortical surface [28]. Surface-based spatial smoothing with a 2 mm full width at half maximum (FWHM) was subsequently performed. A bandpass filter (0.008–0.1 Hz) was then applied, and six head motion parameters were regressed out. Despiking and motion scrubbing were not performed.

We applied a standardized preprocessing pipeline of the HCP-D dataset to the ABIDE dataset. The ABIDE-I and II datasets were preprocessed using QuNex, which adapts HCP preprocessing pipelines [28] for broader applications [29]. In the ABIDE dataset, the T1w structural MRI data were bias-field corrected, skull stripped, and registered to the MNI space. The gray and white matter were segmented, and the cortical surfaces were reconstructed to produce individual anatomical segmentations of both the cortical and subcortical regions [30]. The BOLD time-series data were slice-timing-corrected, head-motion-corrected, and registered to the MNI space. Cortical surface models for the pial and white matter boundaries, along with segmentation masks for subcortical voxels, were subsequently used to generate the grayordinate space [28]. The preprocessed cortical BOLD data were then mapped to the standard gray ordinate space, using a cortical ribbon-constrained volume-to-surface mapping algorithm. Surface-based spatial smoothing with a 2 mm FWHM was performed. Finally, a bandpass filter with a range of 0.008–0.1 Hz was applied, and six head-motion parameters were further regressed out. Despiking and motion scrubbing were not performed.

### Functional connectivity gradients

The present study focused primarily on the first principal gradient, which represents the sensory-DMN axis. We estimated cortex-wide functional connectivity gradients from preprocessed BOLD data using the Schaefer atlas [31] with 200 parcels. Functional connectivity matrices were computed by calculating the Pearson’s correlations between the time-series data of distinct brain regions for each participant. Fisher’s r-to-z transformation was subsequently applied to normalize the distribution of the correlation values. Next, the functional connectivity matrix was refined by retaining only the strongest 10% of the connections per row by following previous studies on functional connectivity gradients [7, 9, 32–34]. An affinity matrix capturing the similarity in connectivity patterns across the cortical areas was subsequently constructed using a normalized angle kernel. Low-dimensional representations, referred to as gradients, were extracted from this matrix by using diffusion map embedding [35] for each participant. A group-level connectivity matrix for the HCP-D datasets was created by averaging the individual functional connectivity matrices, while a group-level template gradient was computed from the averaged functional connectivity matrix. The individual gradients of the HCP-D and ABIDE datasets were aligned to a group-level template gradient using Procrustes rotation [36]. The entire gradient estimation process was conducted using the BrainSpace toolbox [37]. Finally, to mitigate scanner and site effects within the ABIDE dataset, combat harmonization was applied to the gradient values [38].

### Normative modeling

Normative models were constructed using the GAMLSS model [24, 39], which allowed us to capture nonlinear age-related patterns of cortical hierarchy in TD individuals and identify deviations in ASD relative to this normative pattern. This approach has been widely applied across various fields to approximate developmental trajectories from cross-sectional data [22, 40, 41]. Fitting the GAMLSS model involves a four-parameter distribution modeling µ, σ, ν, and τ; corresponding to the mean, variance, skewness, and kurtosis of the feature distribution, respectively. We used the four-parameter sinh-arcsinh (SHASH) distribution [42], following the recommendations for the normative modeling of neuroimaging data [43]. Specifically, region-wise normative modeling of gradient values based on age and the interaction between age and sex was performed as follows:

1 $$\begin{array}{*{20}{c}}{y\, \sim \,SHASH(\mu,\,\sigma,\,\nu,\,\tau ),} \\ {\begin{array}{*{20}{c}}{\mu \, = \,{\beta _\mu }\, + \,{\beta _{\mu \:,age}}f(age)\, + \,{\beta _{\mu \:,age*sex}}f(age*sex),} \\ {\log (\sigma )\, = \,{\beta _\sigma }\, + \,{\beta _{\sigma,age}}f(age),\:} \\ {\log (\nu )\, = \,{\beta _\nu },} \\ {\log (\tau )\, = \,{\beta _\tau },} \end{array}} \end{array}$$

where $\:f(x)$ is a nonlinear function (i.e., P-spline) of $x$. GAMLSS model fitting was implemented using the *gamlss* R package [39]. A normative model for the gradient values was constructed using the HCP-D dataset and then transferred to the TD group of the ABIDE dataset. For the ABIDE dataset, the intercepts (i.e., $\:{\beta _{\mu \:,{\text{new}}}}$, $\:{\beta _{\sigma \:,{\text{new}}}}$) were re-estimated while retaining the nonlinear effect of age fixed [43]. This procedure adjusts the intercept and standard deviation of the model to better correspond to the data from a new dataset while preserving the estimated nonlinear age-related terms. Significant age effects were determined from the $\:{\beta _{\mu \:,{\text{age}}}}$, while multiple comparisons across the brain regions were corrected using the false discovery rate (FDR) procedure [44].

To quantify the extent to which an individual deviates from normative trajectories, we calculated centile scores, which serve as age- and sex-specific indicators of deviations in gradient values across developmental stages. The centile score was determined by comparing each individual’s brain features (i.e., gradient value) with those from the normative distribution. This difference determines an individual’s position within the normative distribution, with higher or lower centile scores indicating the degree of atypicality relative to the reference group. Extreme scores indicate a greater deviation from normal. We utilized ABIDE-I data for the primary normative modeling analyses, and the reproducibility was assessed using ABIDE-II data, following the same preprocessing steps, modeling framework, and exclusion criteria.

### Computing the hierarchy score

We devised a functional hierarchy score as a summary metric (i.e., a scalar value for the whole brain), representing the relative closeness of individual data points with respect to the normative cortical functional hierarchy. We defined this as the cosine similarity between an individual’s gradient ($\:G1$) and the normative gradient for their age ($\:NormG{1}_{age}$), as estimated by the GAMLSS model.

2 $$\:{\text{Hierarchy}}\:{\text{score}} = \frac{{NormG{1_{age}} \cdot \:G1}}{{\left| {\left| {NormG{1_{age}}} \right|} \right|\left| {\left| {G1} \right|} \right|}}$$

This metric assesses how closely the individual gradient aligns with the normative functional hierarchy, reflecting the proximity of the functional hierarchy based on the participants’ age and sex. The higher the score, the closer the individual’s brain matches the normative functional hierarchical pattern. To construct developmental trajectories of hierarchy scores for the ASD and TD groups, we further modeled the age-related changes in hierarchy scores for each group using the GAMLSS from Eq. 1.

### Measures of functional integration and segregation

To assess the topological characteristics of the cortical hierarchy in individuals with ASD, we calculated the participation coefficient (PC), which assesses the integration and segregation of functional networks [45]. A higher PC indicates greater integration and a lower PC indicates greater segregation. The PC of node $\:i$ is defined as follows:

4 $$P{C_i} = 1 - \sum\nolimits_{m \in M} {{{\left( {\frac{{{k_i}\left( m \right)}}{{{k_i}}}} \right)}^2},} $$

where $\:M$ represents the set of network modules, $\:{k_i}(m)$ is the degree of node $\:i$ with respect to all nodes in module $\:m$, and $\:{k}_{i}$ is the total degree of node $\:i$ with respect to all the other nodes in the entire network. PC was calculated from a binarized functional connectivity matrix that retained only the top 10% of positive connections using the Yeo-7 functional network as the module [46]. To control for age, sex, and diagnosis labels, we calculated the PC centile scores based on the normative models specific to each group using the GAMLSS from Eq. 1.

### Mediation analysis

We assessed whether the relationship between the diagnostic group and the hierarchy score was mediated by connectome-level observations. Specifically, a mediation analysis was performed to examine whether the association between the diagnostic group and the hierarchy score was mediated by the whole-brain and network-specific PC centilescores. The significance of the mediation effect was determined through 5,000 bootstraps using *lavaan* package in R [47]. All regression weights were standardized.

### Between-group comparison in functional hierarchy

We evaluated abnormal development of the functional hierarchy (i.e., gradient values or centile scores) in individuals with ASD relative to TD individuals. First, to compare the gradient values between groups, we fitted cortex-wide linear models implemented in the BrainStat toolbox [48], after controlling for age and sex. We further compared the centile scores from the GAMLSS models between the ASD and TD groups using two-sample t-tests. We further computed the magnitude of between-group differences using t-statistics to compare gradient values or centile scores. Multiple comparisons across brain regions were corrected using the FDR procedure [44].

### Replication and behavioral analyses

We replicated our findings using the independent ABIDE-II dataset to verify the reproducibility. We repeated the entire analysis by substituting ABIDE-I with ABIDE-II. Additionally, we correlated social responsiveness scale (SRS) scores with the hierarchy scores [49] to assess the clinical relevance of the scores (Supplementary Tables 1 and 2). For the correlation analysis, we used a subset of the data (ABIDE-I: *n* = 240, ABIDE-II: *n* = 538) to capture the spectrum of autistic traits across both ASD and TD populations. Note that the SRS subdomain scores were only available for the ABIDE-II dataset. Lastly, to assess the impact of uneven age distribution on developmental trajectory shapes in the ABIDE-II replication dataset (Supplementary Fig. 2), we divided the age range into five equal intervals and uniformly sampled participants from each interval. Since this adjustment resulted in some intervals lacking female participants, we simplified the GAMLSS model by removing the interaction term ($\:age\:*\:sex$). We repeated the resampling procedure 100 times and averaged the resulting trajectories. This approach enabled us to assess the stability of the trajectory shapes under a more balanced age distribution (Supplementary Fig. 3A).

### Replication in the longitudinal data

To further validate the cross-sectional findings and examine whether the observed age-related patterns persist within a longitudinal framework, we conducted an additional analysis using the ABIDE-II longitudinal dataset (*n* = 38; Supplementary Table 3). This dataset included repeated neuroimaging measures from participants with ASD and TD at baseline and follow-up, enabling the exploration of developmental trajectories within individuals over time. We employed a GAMLSS with subject-specific random intercepts to incorporate longitudinal correlations and individual-level variability. We did not consider age-sex interaction due to the limited sample size. The model parameters µ, σ, ν, and τ included subject-specific random effects (γ) to account for inter-individual differences:

4 $$\begin{array}{*{20}{c}}{\begin{array}{*{20}{c}}{y\, \sim\,(\mu,\sigma,\nu,\tau ),} \\ {\mu\, = \:{\beta _\mu }\, + \,{\beta _{\mu,age}}f(age) + \gamma,} \\ {\log (\sigma )\, = \,{\beta _\sigma }\, + \,{\beta _{\sigma,age}}f(age) + \gamma,\:} \\ {\log (\nu)\, = \,{\beta _\nu }\, + \,\gamma,} \end{array}} \\ {\log (\tau)\, = \,{\beta _\tau }\, + \,\gamma,} \end{array}$$

where γ represents the random intercepts for each subject for the four parameters. This modeling approach allowed us to estimate age-related changes within a longitudinal framework, taking into account for the repeated observations from the same individuals.

### Sensitivity analyses

Multiple sensitivity analyses have been conducted to demonstrate the robustness of our findings. First, we performed the analysis using a multimodal parcellation (i.e., Glasser atlas) [50] to assess whether the findings could be replicated with different parcellation schemes. We correlated the spatial patterns of centile scores between the Schaefer and Glasser atlases on the conte69 surface. Second, we conducted analyses using connectivity matrices with different threshold levels. We replicated the main findings using 5% and 20% thresholds and spatially correlated the centile scores of ASD of different thresholds with those from the 10% threshold. Third, the dataset included left-handed, right-handed, and ambidextrous participants (Supplementary Tables 1–3). Excluding participants with ambidextrous handedness, differences in centile and hierarchy scores between left-handed and right-handed participants were examined. To evaluate the potential impact of handedness, we included handedness as a covariate in the GAMLSS modeling of hierarchy scores as follows:

$$\begin{gathered}\:\mu \: = \:{\beta _\mu }{\mkern 1mu} + {\mkern 1mu} {\beta _{\mu \:,age}}f(age) \hfill \\\,\,\,\,\,\,\, + {\mkern 1mu} {\beta _{\mu \:,age*sex}}f(age*sex){\mkern 1mu} + \,{\beta _{\mu \:,Handedness}}Handedness. \hfill \\ \end{gathered} $$

Fourth, to assess the potential impact of head motion on the results, we incorporated mean FD as a covariate in the GAMLSS in modeling functional gradients and hierarchy scores. Specifically, we modified the model as follows:

$$\begin{gathered}\:\mu \: = \:{\beta _{\mu \:}}\, + \,{\beta _{\mu \:,age}}f(age){\mkern 1mu} \hfill \\\,\,\,\,\,\, + \,{\beta _{\mu \:,age*sex}}f(age*sex) + \,{\beta _{\mu \:,meanFD}}meanFD. \hfill \\ \end{gathered} $$

## Results

### Aberrant development of functional hierarchy in autism

**Fig. 1:**
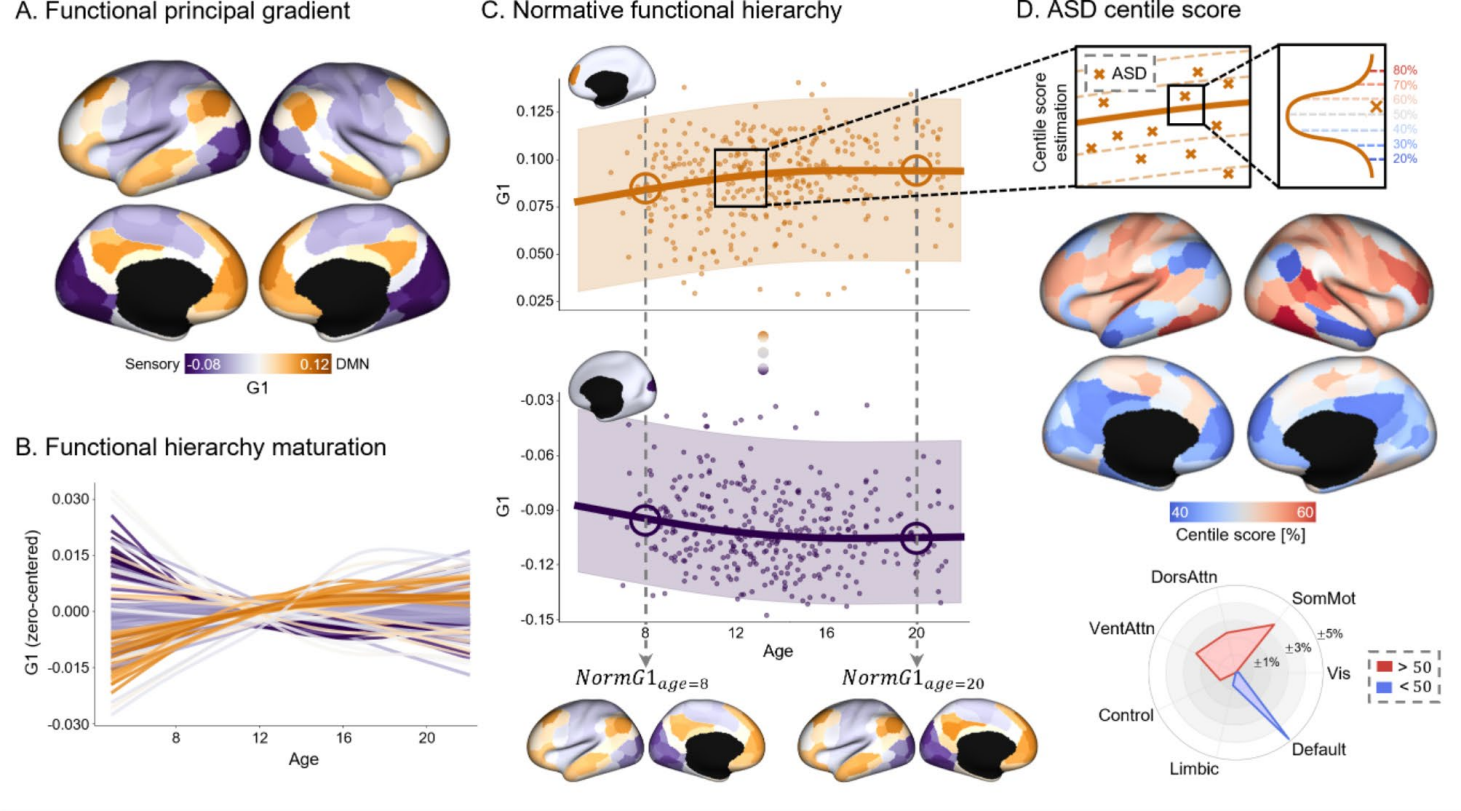
Atypical development of the cortical hierarchy in ASD. **(A)** The principal gradients derived from functional connectivity of the TD group using the HCP-D dataset. **(B)** The maturational trajectory of the whole-brain functional hierarchy. Brain regions with significant age effects are plotted ($\:{P}_{FDR}\:<\:0.05$), and the color of each line corresponds to the gradient values from **(A)**. **(C)** Normative functional hierarchy across developmental stages ($\:{Norm}_{age}$). Here, we present two representative regions, one from low-level sensory areas and another from higher-order DMN regions. **(D)** Centile scores relative to the normative trajectory are calculated and shown on the brain surfaces. The centile scores are stratified using canonical functional networks. Abbreviations: TD, typically developing; ASD, Autism spectrum disorder; Vis, Visual; SomMot, Somatomotor; DorsAttn, Dorsal attention; VentAttn, Ventral attention; FDR, False discovery rate

We generated a functional principal gradient using the HCP-D dataset to assess the developmental trajectory of the cortical hierarchy. This represents a continuum along an axis with sensory regions at one end and the DMN at the opposite end (Fig. 1A). When we fitted the functional gradients using the GAMLSS model, 130 of the 200 regions of interest (ROIs) showed significant changes with age (FDR-adjusted p-value ($\:{P}_{FDR}$) < 0.05). The developmental curve of the ROI-level principal gradient with significant age effects revealed an overall gradient expansion maturation, with the gradient values of the sensory regions (*purple*) decreasing, and the gradient values of the DMN regions (*orange*) increasing (Fig. 1B). Based on the normative developmental trajectory, we estimated the whole-brain normative functional hierarchy (i.e., principal gradients) at a specific age ($\:{Norm}_{age}$), positing that the typical functional hierarchy at each developmental stage (i.e., age) was modeled as the median of the fitted distribution from GAMLSS (Fig. 1C).

We subsequently quantified the developmental patterns of the functional hierarchy of individuals with ASD as centile scores related to the normative trajectory. Unlike the classical case-control paradigm, the normative modeling approach allows for a comparison of the abnormal functional hierarchy in ASD in terms of its relative distance from the TD brain. As previous research has pointed out, the principal functional gradient is suppressed in ASD, manifesting by a decrease in the DMN regions of the parietal and temporal lobes and an increase in attention networks (Hong et al., 2019). Herein, we revealed through network-wise stratification that the gradient values of the sensory and attention networks (i.e., somatomotor, dorsal attention, and ventral attention) increased, while there was a pronounced decrease in the DMN (Fig. 1D). The t-statistics from the case-control comparison of centile scores and the principal gradients revealed similar patterns across networks. This alignment of the spatial distribution of significant regions and network-level summary results validated the clinical sensitivity of centile scoring (Supplementary Fig. 4). When we examined the second gradient (G2), which represents the sensorimotor-visual axis, we observed similar patterns of centile scores, with slightly reduced effects in the DMN and increased effects in sensory regions (Supplementary Fig. 5).

### Hierarchy score analysis

**Fig. 2:**
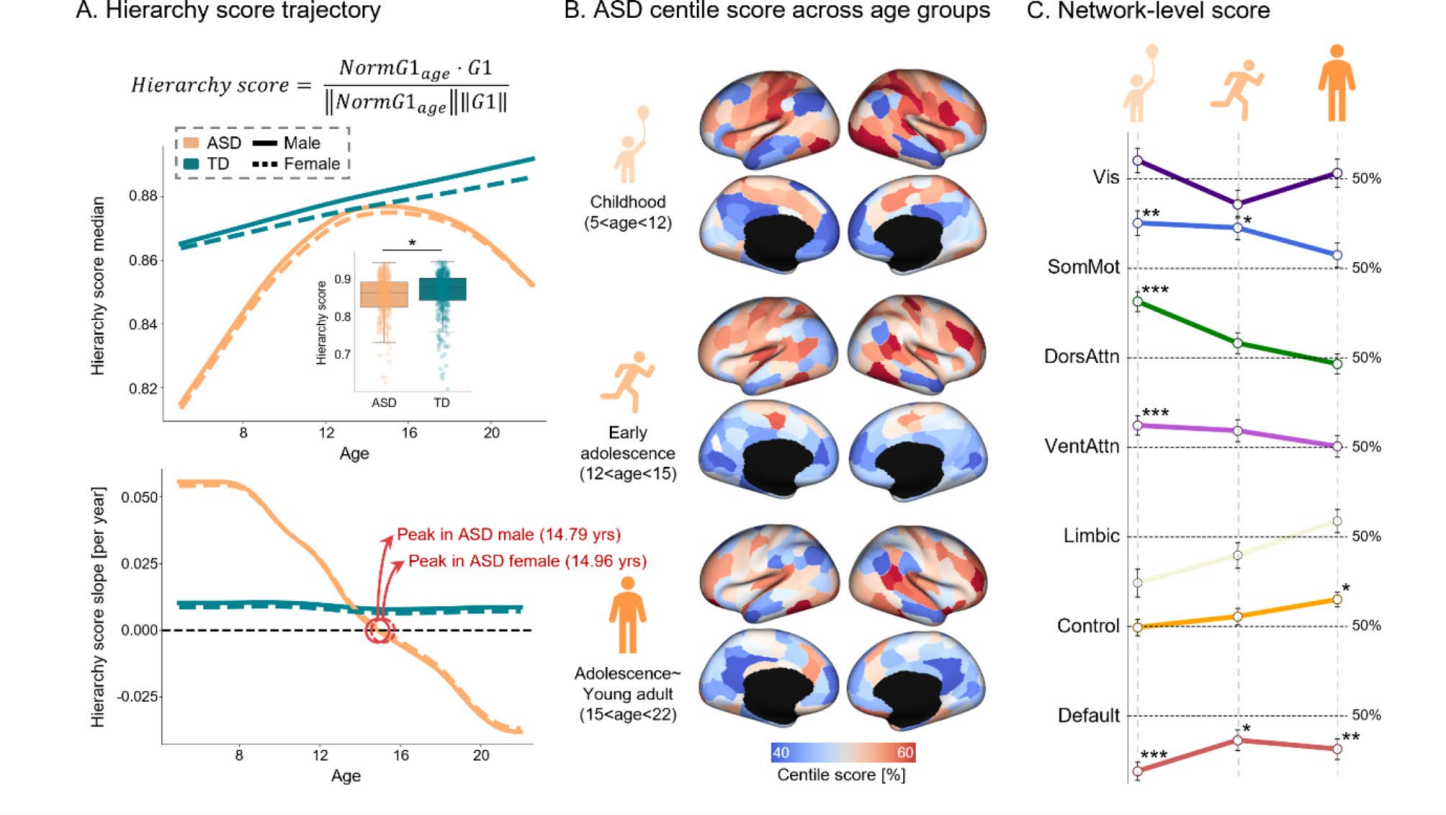
Hierarchy score analysis. (**A)** Calculation of the hierarchy scores and the trajectory of median and slope values [per year]. **(B)** Whole-brain centile scores across three developmental stages. **(C)** Network-level stratification of whole-brain centile scores across different developmental stages. ∗, *p*<0.05; ∗∗, *p*<0.01; ∗∗∗, *p*<0.001. Abbreviations: TD, typically developing; ASD, Autism spectrum disorder; Vis, Visual; SomMot, Somatomotor; DorsAttn, Dorsal attention; VentAttn, Ventral attention

To succinctly compute the degree of atypical maturation of ASD, we estimated an individual-level summary score (i.e., hierarchy score), and assessed the developmental trajectory of each group separately using the GAMLSS. We initially tested for group differences in the hierarchy score, identifying a significant decrease in the ASD group (*p* < 0.05, t = -2.536), indicating improved immaturity of the cortical hierarchy. Next, we explored the developmental trajectory underlying compromised functional hierarchy in individuals with ASD (Fig. 2A). While the group-level hierarchy score of TD individuals increased at a nearly constant rate (slope: 0.009/year $\:\pm\:$ 0.001/year for males and 0.0077/year $\:\pm\:$ 0.001/year for females), that of individuals with ASD showed a markedly distinct developmental trajectory. In early development, ASD individuals exhibited a steeper increase (slope: 0.013/year $\:\pm\:$ 0.033/year for males and 0.013/year $\:\pm\:$ 0.032/year for females), catching up to the typical cortical hierarchy by around 14.78 years for males and 14.98 years for females. Following this peak, the hierarchy score for the ASD group began to decline in the remaining age groups.

These results underscore the temporal heterogeneity within the cortical hierarchy. To better understand this heterogeneity, an age-bin analysis was conducted, in which we grouped the study participants into three developmental stages based on age, following the classifications used in prior research [51, 52]: childhood (5–12 years; ASD = 110, TD = 125), early adolescence (12–15 years; ASD = 101, TD = 121), and adolescents and young adults (15–22 years; ASD = 122, TD = 123) (Fig. 2B and **C**). Notably, each stage exhibited a unique whole-brain centile score pattern. During childhood, higher gradient values were predominantly found in the somatomotor ($\:t=3.43,\:p=0.001$), dorsal attention ($\:t=4.78,\:\text{p}<$1e-4), and ventral attention networks ($\:t=4.27,\:\text{p}<$1e-4), whereas lower scores were primarily observed in the DMN ($\:t=-5.67,\:\text{p}<$1e-6). Significant network-level alterations were also found in both low-level and higher-order networks, making childhood the most atypical stage in terms of cortical hierarchy development. In early adolescence, network-level significance diminished, except for the somatomotor network ($\:t=2.62,\:p=0.035$) and DMN ($\:t=-2.86,\:p=0.035$). However, as they entered late adolescence and young adulthood, deviations in the cortical functional hierarchy were predominantly driven by decreased gradient values in the precuneus and posterior cingulate cortex within the DMN ($\:t=-3.72,\:p=0.002$) and increased values in the control network ($\:t=2.88,\:p=0.016$). These findings indicate that during early development, both low-level and higher-order networks contribute to the atypical cortical hierarchy in ASD. However, lower-level networks tend to follow a typical developmental trajectory throughout development, whereas higher-order networks continue to lag, resulting in persistent abnormalities. Similarly, G2 exhibited a hierarchy pattern with a nonlinear trajectory (Supplementary Fig. 6). Although the overall group differences in G2-based hierarchy scores were not statistically significant (t = − 1.366, *p* = 0.172), G2 showed stronger differences in sensory areas, whereas G1 revealed more pronounced changes in DMN regions. These findings extend our understanding of ASD beyond the primary sensory–transmodal hierarchy to sensorimotor–visual boundaries.

Additionally, we analyzed the heterogeneity of the hierarchy score across age by assessing the temporal dynamics of sigma ($\:\sigma\:$, variance) in the GAMLSS model. Hierarchy score trajectories with the 2.5% and 97.5% centiles revealed that the TD group showed a narrowing trend with age, whereas the ASD group did not follow this pattern (Supplementary Fig. 7A). This observation was corroborated by the sigma analysis, which revealed a significant effect of age only in the TD group (Supplementary Fig. 7B). Specifically, sigma decreased significantly in the TD group ($\:t=-2.415,\:p=0.016$), indicating that individuals increasingly conformed to norms throughout development. In contrast, this remained nearly constant in the ASD group ($\:t=0.047,\:p=0.96$), indicating a lack of convergence among individuals with ASD.

### Topological characteristics underlying cortical hierarchy

**Fig. 3:**
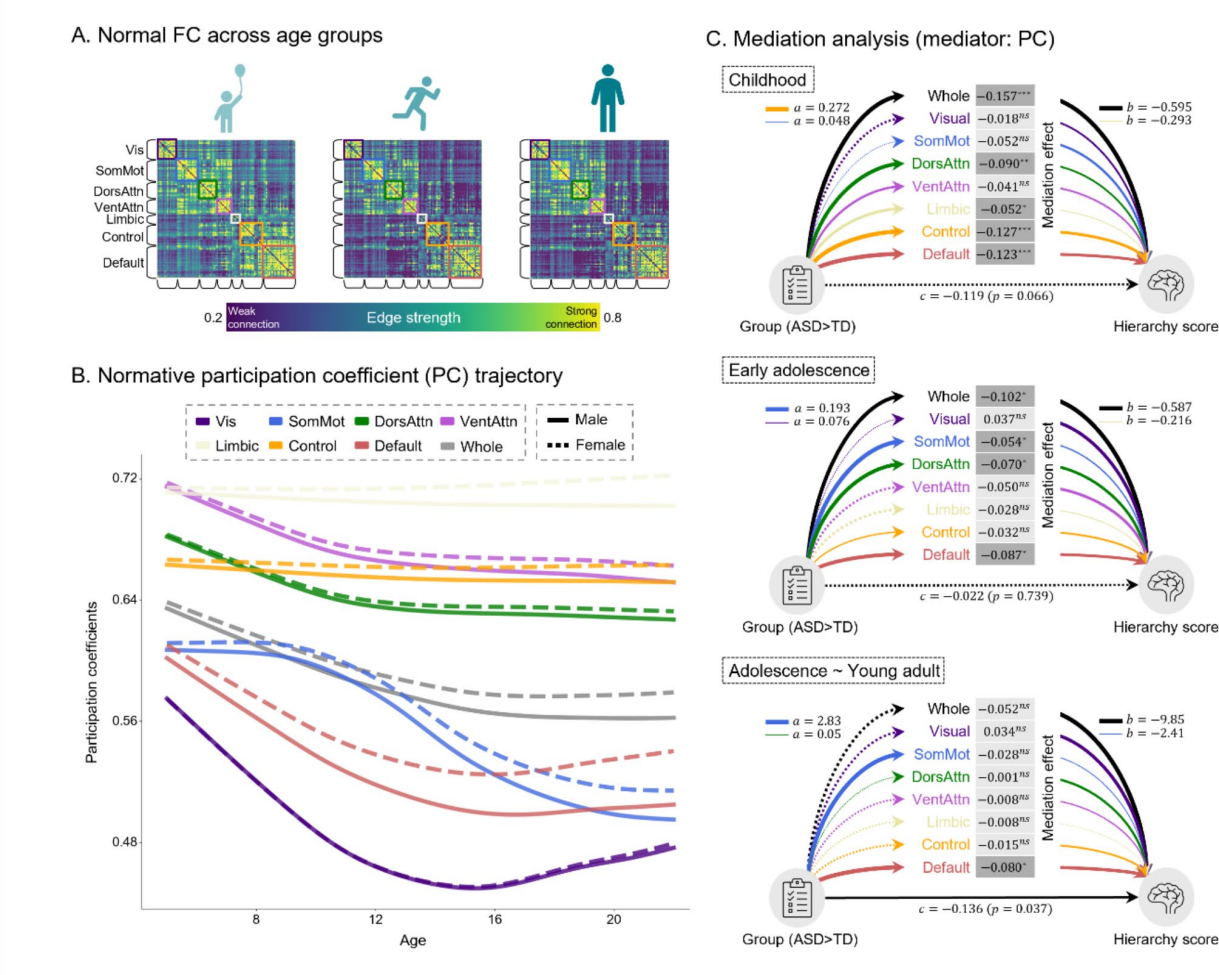
Topological characteristics during development. **(A)** FC of the TD group across different developmental stages. To improve visibility, each network within the FC matrix is outlined by the representative network colors shown in the legend for B. **(B)** The normative trajectory of whole-brain and network-wise PC. **(C)** Results of mediation analysis for each stage. The paths *a*, *b*, and *c* denote direct effects between two variables. Significant paths are indicated as solid lines. The thickness of the lines corresponds to the magnitude of the effect, with the largest and smallest effects being annotated. Significant mediation effects are indicated by darker boxes and asterisks (∗, *p*<0.05; ∗∗, *p*<0.01; ∗∗∗, *p*<0.001; *ns*, not significant). Abbreviations: FC, functional connectivity; TD, typically developing; PC, participation coefficient; Whole, whole-brain; Vis, Visual; SomMot, Somatomotor; DorsAttn, Dorsal attention; VentAttn, Ventral attention

Herein, we investigated the topological characteristics underlying atypical cortical hierarchies across the developmental stages in individuals with ASD. In the TD group, the most pronounced change was a decrease in sensory-transmodal edge strength, indicating segregation between the two extremes along the cortical hierarchy (Fig. 3A). To evaluate the functional segregation and integration patterns of the connectome, we characterized the normative trajectory of the PC by fitting other whole-brain and network-wise GAMLSS models (Fig. 3B). All models, except the limbic and control networks, showed significant decreases in PC with age ($\:{P}_{FDR}<\:0.05$). Notably, the PC decline in the visual and somatomotor networks and DMN was pronounced, reflecting typical maturation of the functional hierarchy.

To provide a potential mechanistic explanation for the effect of connectome changes on the altered hierarchy, we performed a mediation analysis. Specifically, we tested whether whole-brain and network-wise PC centile scores mediated group differences in the hierarchy scores. We initially evaluated the mediation effects at each developmental stage (Fig. 3C). The analysis revealed two key findings: (1) the number of networks showing significant mediation effects and their effect sizes decreased as developmental stages progressed, and (2) reduced segregation within the DMN emerged as the only mediator of the reduced hierarchy score in adolescence and young adulthood. These results are consistent with the developmental changes in the centile scores (Fig. 2C), indicating that both the sensory and transmodal regions are key markers during early developmental stages, while the higher-order DMN is a notable marker during adolescence and young adulthood in ASD. Additionally, we performed analysis on all participants, rather than dividing the developmental stages, finding that all PC centile scores, except the visual network, significantly mediated the change in the hierarchy score in ASD (Supplementary Fig. 8).

### Replication analysis

To confirm the generalizability of our study, we evaluated the replicability of our findings using independent samples from the ABIDE II dataset. First, the maturation pattern of the principal functional gradient was replicated with an expanding sensory-DMN axis (Supplementary Fig. 9A and B). Moreover, consistent decreases in the principal gradient values within the DMN and increases in the sensory and attention networks were observed (Supplementary Fig. 4C). We next examined the trajectory of the hierarchy score to test whether the unique developmental trajectory identified in the discovery sample could be reproduced (Supplementary Fig. 10A). Overall, the TD group showed a gradual increase in the hierarchy scores, whereas the ASD group exhibited a sharp increase, catching up with their initially lower scores. However, the trajectory lacked an early adolescent peak, followed by a decline in the discovery dataset, presumably due to the sparse sample density after childhood (Supplementary Fig. 2). After adjusting the age distribution in the ABIDE-II dataset, the average trajectory retained its nonlinear, inverted U-shaped pattern (Supplementary Fig. 3B). These findings indicate that the observed linear patterns in the replication dataset were likely influenced by sampling imbalances. Finally, the centile score across developmental stages reaffirmed our key finding that both low-level and higher-order networks were impaired during childhood, with only abnormalities in the higher-order network remaining significant as children matured (Supplementary Fig. 10B and 10 C).

The longitudinal analyses using the ABIDE-II longitudinal dataset supported our cross-sectional findings. We identified an inverted U-shaped association between age and the hierarchy score in the ASD group, closely mirroring the patterns observed in the cross-sectional sample (Supplementary Fig. 11). Notably, the age-related curves remained consistent across repeated measurements within individuals, suggesting that the observed developmental trajectories reflect stable population-level trends rather than idiosyncratic sampling or cross-sectional artifacts. While some nuances emerged, such as slight variations in the peak associations, the overall profile of age-related changes remained similar. These longitudinal findings confirm that the patterns detected in the cross-sectional data are not solely attributable to between-subject variability or cohort effects.

### Relevance to symptom severity

**Fig. 4:**
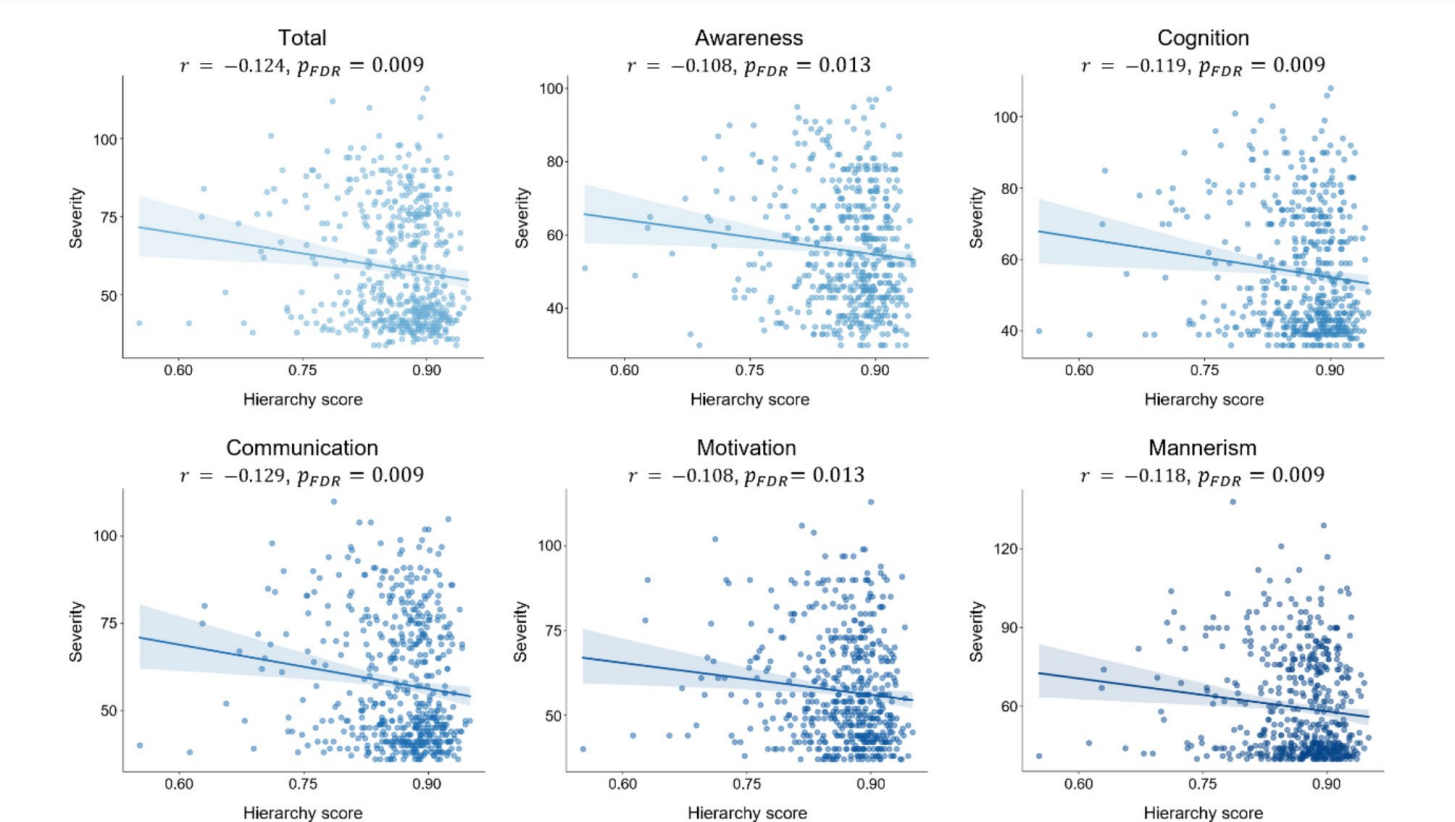
Correlations between the hierarchy score and symptom severity. Scatter plots between the hierarchy score and total and five subscores of the social responsiveness scale (SRS). Abbreviations: SRS, social responsiveness scale; r, Pearson correlation coefficient; $\:{p}_{FDR}$, FDR corrected *p*-value

We next evaluated the clinical relevance of the hierarchy score by correlating it with the severity of ASD symptoms. We assessed the total SRS score and five subscores: awareness, cognition, communication, motivation, and mannerisms (Fig. 4). Our findings revealed significant correlations across the total and all five subscores, with an average correlation of -0.118 ± 5.96e-5, where the highest correlation was observed in the communication subdomain. These results indicate that the hierarchy score effectively reflects the severity of autistic symptoms, such that lower hierarchy scores, indicating higher deviations from the normative cortical hierarchy, are associated with higher symptom severity. In ABIDE-I dataset, the effect was not significant (*r* = − 0.094, *p* = 0.145; Supplementary Fig. 12), and it may be attributable to a smaller sample size.

### Sensitivity analyses

First, when we used the multimodal parcellation scheme [50], we found consistent results with our main findings based on the Schaefer atlas [31]. The principal functional gradient was suppressed in the DMN in ASD (Supplementary Fig. 13), and spatial correlations of the centile scores were notably high (childhood: *r* = 0.769, pspin = 0.006, early adolescence: *r* = 0.698, pspin < 1e-3, and adolescence and young adult: *r* = 0.655, pspin < 1e-3; Supplementary Fig. 14). These findings indicate that our results are robust and can be replicated across different parcellation schemes. Second, the different functional connectivity thresholds did not change the findings. We observed consistent principal gradient patterns of the sensory-DMN axis in both thresholds and high spatial correlations in centile score across developmental stages (5%: *r* = 0.870 ± 0.03; 20%: *r* = 0.883 ± 0.06; Supplementary Figs. 15 and 16). Third, our analysis did not identify any parcels with significant differences in centile or hierarchy scores between left- and right-handed participants (Supplementary Fig. 17). When we tested $\:{\beta _{\mu \:,Handedness}}$ in the GAMLSS modeling, no measurable effect was found in both ASD and TD groups (ASD: *p* = 0.284, TD: *p* = 833). These findings suggest that handedness did not influence the findings in our study. Fourth, consistent with the main findings, the hierarchy score remained significantly different between the TD and ASD groups (t=-2.372, *p* = 0.018), even after controlling for mean framewise displacement. Including head motion as a covariate in the GAMLSS model also revealed nonlinear trajectories, with high spatial correlations of centile scores with the original model observed across the three developmental stages (*r* = 0.976 ± 0.02; Supplementary Fig. 18).

## Discussion

An increasing body of neuroimaging research has highlighted the age-related abnormalities in the brain connectome organization along the cortical hierarchy in individuals with ASD [19, 53, 54]. In the present study, we expanded upon these observations by examining population-level age-related associations of the cortical hierarchy in ASD using principal functional gradients and advanced GAMLSS analyses. Although our primary analyses were cross-sectional, we employed a normative modeling approach to approximate developmental trajectories at the population level. To strengthen our interpretation, we reinforced the findings using a smaller subset of the ABIDE-II dataset with longitudinal measures, providing preliminary confirmation that the observed patterns are not solely artifacts of cross-sectional sampling. Distinct variations were observed in the ASD group by tracing the developmental trajectory of cortical hierarchy. Unlike the gradual maturation of the hierarchy seen in TD individuals, the ASD group followed a non-linear trajectory, characterized by delayed development in childhood, followed by a rapid “catch-up” phase during adolescence. Topological analysis revealed that altered segregation patterns in the sensory and DMN regions significantly mediated group differences in cortical hierarchy. Finally, associations with symptom severity confirmed the relevance of the hierarchy scores in describing autism-related symptomatology.

Extensive studies have investigated the atypical development of functional connectomes in individuals with ASD. For example, some studies have shown that children with ASD exhibit increased local connectivity and reduced long-range connectivity [55], whereas others have reported diminished connectivity among older adolescents and adults with ASD [56]. However, the field has yet to reach a consensus on the specific patterns of connectome reorganization in ASD, likely due to the heterogeneity of the condition, with factors such as age and sex being the major contributors [57, 58]. One approach to disentangling this heterogeneity is clustering, which identifies subtypes within ASD [8, 20, 59]. Although clustering has been successful in revealing the distinct topological and behavioral characteristics of ASD, technical challenges remain, such as defining the optimal number of clusters [60] and quantifying the individual-level brain features [61, 62]. Another approach to explain the heterogeneity of autism is the normative modeling approach [21, 23], which is well suited for capturing variability by modeling not only the mean, but also higher-order statistical moments, such as the variance, skewness, and kurtosis, of the connectome [24, 43]. As ASD is closely linked to brain maturation, in which significant changes occur during development, identifying atypical developmental trajectories in ASD may capture the individual-level characteristics of individuals with ASD. In the present study, we employed the GAMLSS model, one of the most widely used methods in normative modeling. Unlike the generalized linear model, the GAMLSS models the relationship between the predictors and response variables using nonlinear functions. Additionally, by relaxing the assumption of homoscedasticity, GAMLSS allows for the implicit modeling of heterogeneity induced by demographic factors through higher-order statistical moments. By accounting for the nonlinear developmental trajectories and interactions between age and sex, this model offers a more nuanced understanding of how ASD manifests across different developmental stages.

The developmental trajectory analysis in the present study revealed that the sensory and attention networks exhibited pronounced deviations early in development, contributing to an atypical cortical hierarchy in individuals with ASD. However, as individuals entered adolescence, these low-level networks tended to normalize, whereas higher-order networks, particularly the DMN, remained impaired. This continued dysfunction in the higher-order networks, particularly in the precuneus and posterior cingulate cortex, became more pronounced during the later development. This pattern aligns with the sensory-first hypothesis, which suggests that atypical connectome organization initially emerges in sensory regions during early development, with cascading effects observed in higher-order transmodal regions in later stages [3]. Higher-order transmodal regions, including the DMN, are critical regions in self-referential thought, social cognition, and integration of multisensory information [63, 64]. Previous studies have reported that disruptions in the connectome organization in ASD are strongly linked to risk gene expression across developmental stages [65, 66]. Specifically, thalamocortical connectivity, which links subcortical regions to the frontoparietal and temporal cortices, has been identified as a crucial marker of adult ASD, and is associated with social cognition and communication [65–68]. These findings complement our results, which indicate that different sensory regions are particularly affected in childhood, but tend to normalize in adolescence, whereas the control and DMN regions exhibit abnormalities beginning in adolescence.

Topological analysis offers a complementary perspective, emphasizing the roles of integration and segregation in shaping the cortical hierarchy. While functional segregation between the unimodal and transmodal regions was evident in the TD group, this became diminished in individuals with ASD. Prior studies have also shown that the functional connectome topology is linked to the atypical cortical hierarchy in ASD [7, 66, 69, 70]. Hong et al. demonstrated a marked decrease in gradient values within rich-club nodes connected by long-range connections, coupled with increasing scores in peripheral nodes connected by shorter connections [7]. This pattern indicates reduced segregation between the rich-club core and its periphery in individuals with ASD. These topological disruptions appear closely tied to the first principal gradient, indicating that local topological abnormalities may reflect broader hierarchical imbalances central to ASD’s core challenges. We conducted a mediation analysis to explicitly link functional topology with a hierarchical structure in ASD individuals. Our findings revealed that diminished segregation, characterized by disturbed functional topography in both low-level and higher-order networks, contributes to a compromised cortical hierarchy during childhood. As development progressed, the influence of higher-order networks persisted and intensified, providing a topological framework for understanding the interactions between developmental stages and functional gradients in individuals with ASD.

Cortical hierarchy is linked to a wide range of ASD symptoms [7]. Specifically, abnormalities in lower-level networks create a cascading effect that affects higher-order networks and influences behavior [13]. Consistent with previous findings, our research showed that the hierarchy score, which follows a nonlinear developmental trajectory, is significantly associated with symptom severity in ASD. The hierarchy score reflects deviations from typical cortical organization, with lower scores indicating greater abnormality and a stronger relationship with heightened symptom severity. While the score appears to align more closely with normative patterns during early adolescence, atypicalities persist during childhood and young adulthood. This transient alignment during early adolescence may contribute to the observed decrease in symptom severity during this stage. However, the findings caution that symptoms could re-emerge or worsen during adolescence as cortical hierarchy once again deviates from typical developmental trajectories. This phenomenon, referred to as “compensation” [71], suggests that while neurobiological abnormalities persist, behavioral phenotypes appear to improve temporarily. By illuminating how neurobiological changes relate to symptom progression, our findings highlight the potential of the hierarchy score to help clinicians identify critical developmental windows for intervention. Such insights could ultimately guide personalized strategies to improve long-term outcomes and mitigate symptom exacerbation during later developmental stages [18].

## Limitations

Despite its strengths, this study has several limitations. First, the small sample size, particularly in participants in the older age range, limited the generalizability of our findings, especially regarding the developmental trajectory during late adolescence and young adulthood. Uncertainty in the trajectory significantly increased at age extremes where data points are limited, making it difficult to draw robust conclusions regarding these developmental periods [72]. The linear trajectories observed in the replication dataset (i.e., ABIDE-II) likely arose from age distribution imbalances. Future studies should prioritize datasets with well-balanced age distributions and implement rigorous sensitivity analyses to ensure the reliability and nuanced interpretation of developmental trajectory analyses in ASD research. Although our longitudinal analysis supports the interpretation of age-related associations as developmental trajectories, it is important to acknowledge certain caveats. Longitudinal datasets remain limited in ASD research, and factors such as small sample sizes, short follow-up intervals, and demographic heterogeneity may influence the generalizability of these results. Furthermore, while the presence of longitudinal data mitigates some concerns about inferring development from cross-sectional snapshots, additional large-scale, well-characterized longitudinal cohorts with multiple time points are essential to further refine our understanding of neurodevelopmental changes in ASD. Future research should be extended by including larger and more evenly distributed age groups with longitudinal settings. The use of GAMLSS for modeling nonlinear trajectories also faces inherent challenges, such as potential overfitting and complexity in interpretation, which could affect the robustness of the results [43]. Additionally, expanding this framework to other neurodevelopmental disorders, such as attention deficit/hyperactivity disorder, and schizophrenia, could provide better comparative insights into disruptions in the cortical hierarchy across different conditions. Moreover, integrating genetic and molecular data into these models could offer a more comprehensive view of how aberrant neurobiological mechanisms contribute to functional abnormalities observed in ASD.

## Conclusions

In conclusion, our findings provide insight into the developmental trajectory of the cortical hierarchy in ASD, thus highlighting distinct maturation patterns and persistent dysfunction in higher-order networks. Early deviations in sensory networks followed by long-term abnormalities in the DMN further underscore the heterogeneous nature of ASD. By extending these findings to connectome topology, we demonstrated how network integration and segregation patterns contribute to the atypical development of the cortical hierarchy. The observed relationships between the hierarchy scores and behavioral outcomes suggests that alterations in the cortical hierarchy may serve as key markers to identify the severity and progression of ASD. Collectively, our study contributes to the growing body of evidence indicating that an atypical cortical hierarchy is a hallmark of ASD, with potential implications for early diagnosis and targeted interventions. Future research exploring the broader neurodevelopmental landscape will help refine our understanding of these mechanisms and their impact on long-term outcomes.

## Electronic supplementary material

Below is the link to the electronic supplementary material.

Supplementary Material 1

## Data Availability

The imaging and phenotypic data were provided by the Autism Brain Imaging Data Exchange initiative (ABIDE-I and II; https://fcon_1000.projects.nitrc.org/indi/abide/). The HCP-Development Lifespan 2.0 release is obtained from the HCP (https://www.humanconnectome.org/).
