# Supplementary material for: Atypical maturation of the functional connectome hierarchy in autism
Source: Mol Autism. 2025 Mar 26;16:21. doi: 10.1186/s13229-025-00641-9 (PMC11948645; doi:10.1186/s13229-025-00641-9)
Supplement: Supplementary file 1 — Supplementary Material 1 [file 13229_2025_641_MOESM1_ESM.docx]

**Supplementary Information**


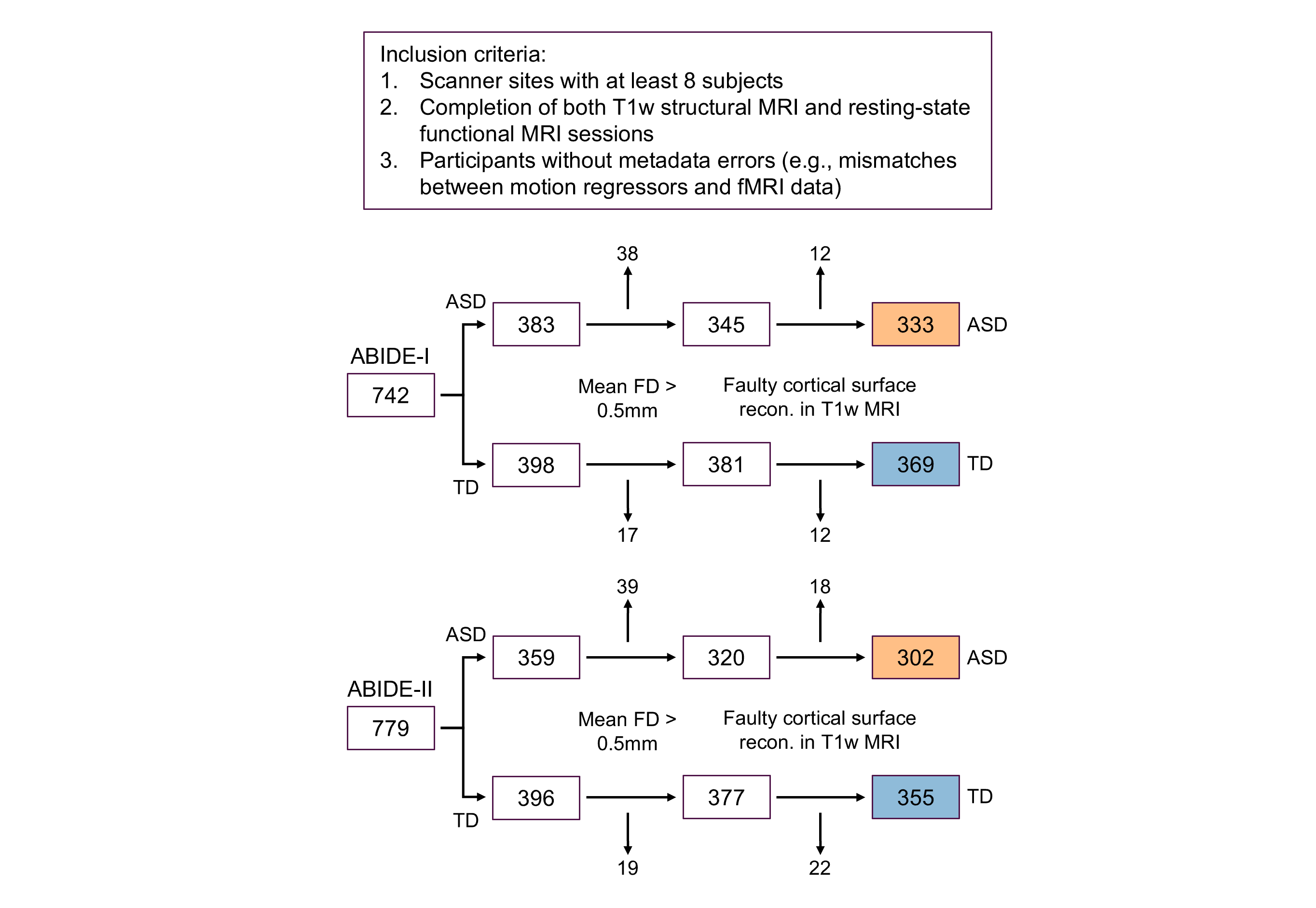


**Supplementary Fig. 1 | Flowchart for the participant selection procedure for the ABIDE cohorts.**

Abbreviations: MRI, magnetic resonance imaging; ABIDE, Autism Brain Imaging Data Exchange; FD, framewise displacement; T1w, T1-weighted; TD, typically developing; ASD, Autism spectrum disorder.

**
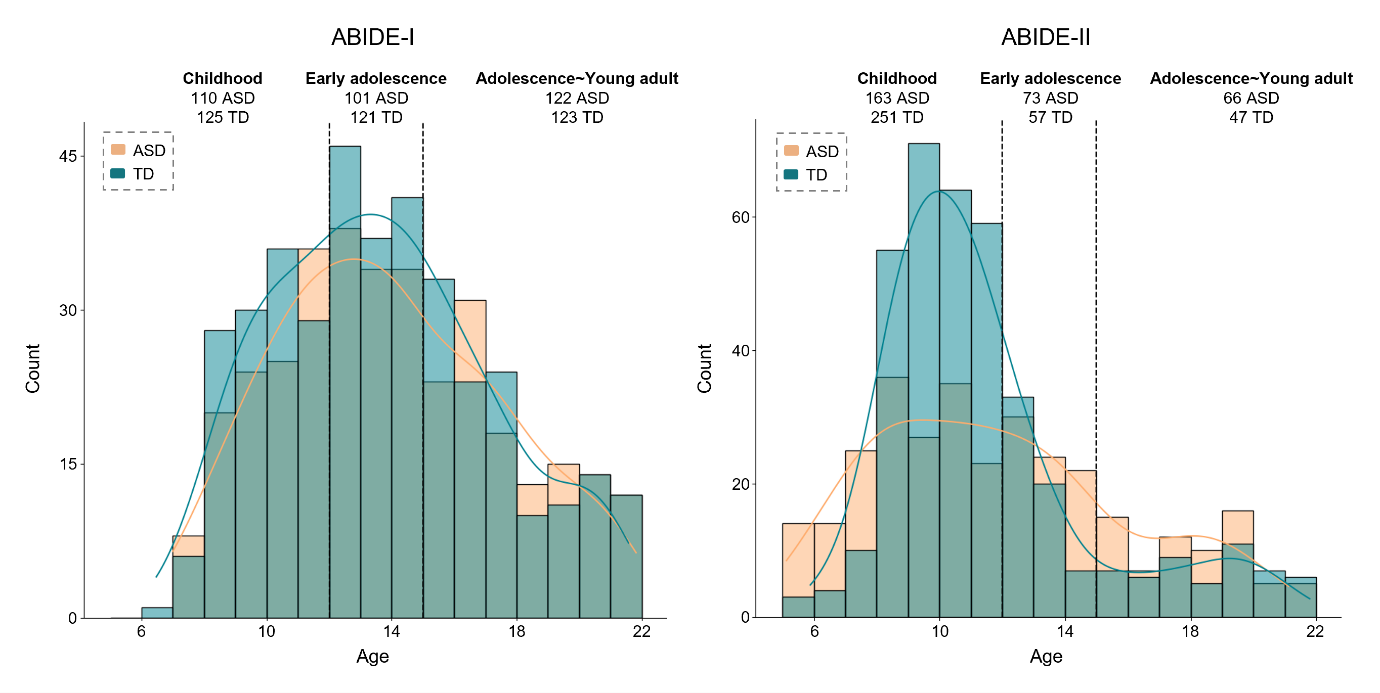
Supplementary Fig. 2 | Age distribution of the discovery (ABIDE-I) and replication (ABIDE-II) datasets.** We noted a significant difference in age distribution between the two datasets (two-sample Kolmogorov-Smirnov test; p < 0.001). The replication dataset was found to have a larger proportion of subjects in childhood, while the discovery dataset presents a more balanced distribution across different developmental stages.
Abbreviations: TD, typically developing; ASD, Autism spectrum disorder; ABIDE, Autism Brain Imaging Data Exchange.


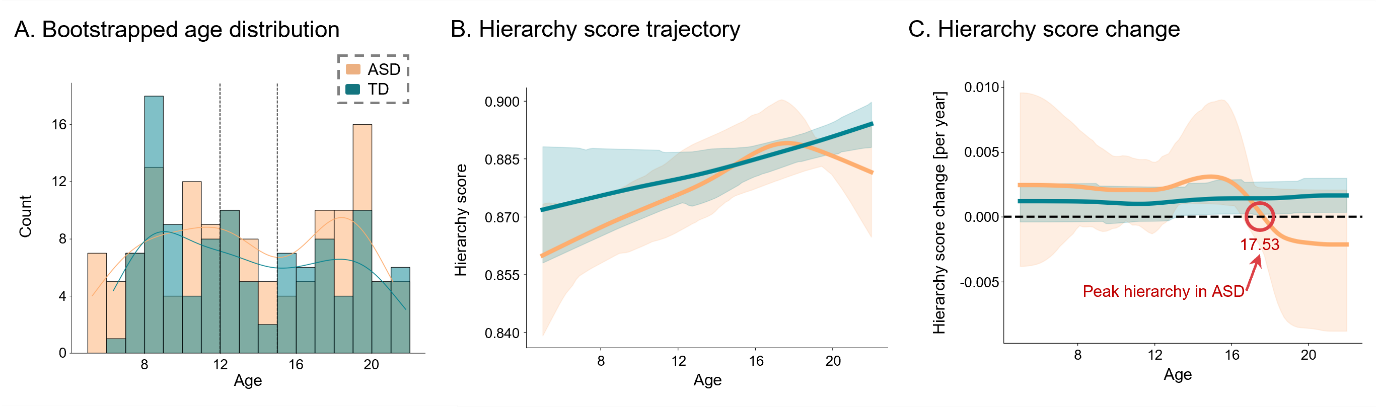


**Supplementary Fig. 3 | Sensitivity analyses after adjusting the age distribution in the ABIDE-II dataset.** **(A)** Age distribution from a single representative sample among the 100 resampled data. **(B)** The average trajectory curve, retaining nonlinear characteristics under the adjusted age distribution, is shown along with confidence intervals derived from 100 curves. **(C)** Slope values [per year] are presented with confidence intervals calculated from the same 100 curves.


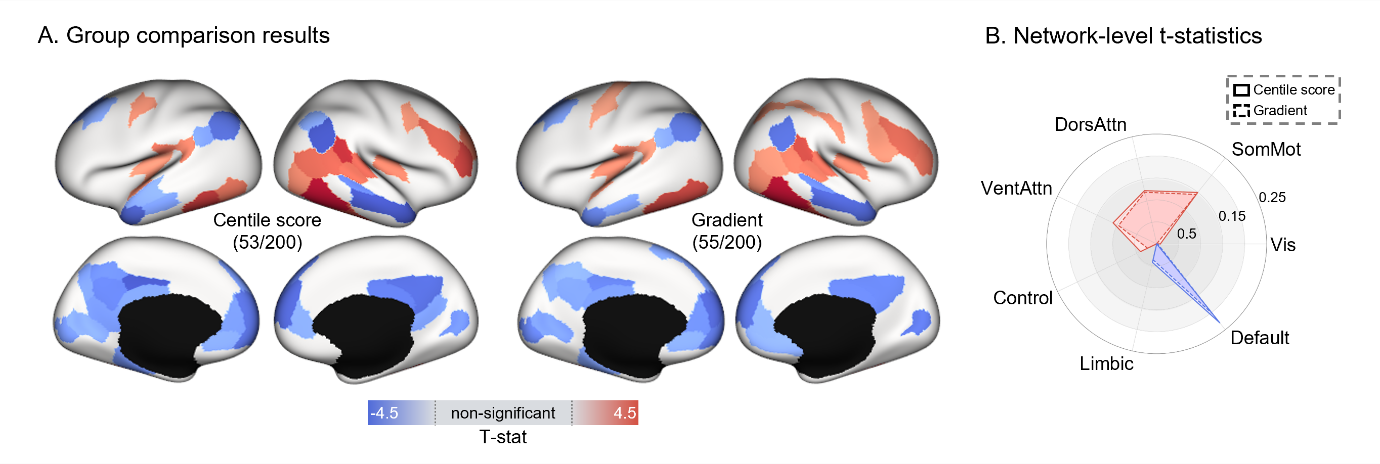


**Supplementary Fig. 4 | Comparison of the effect sizes between the centile score and gradient. (A)** Whole-brain statistics obtained from group comparisons using centile scores and original functional gradients. **(B)** Stratification of t-statistics at the canonical functional network level.
Abbreviations: Vis, Visual; SomMot, Somatomotor; DorsAttn, Dorsal attention; VentAttn, Ventral attention; Whole, Whole-brain.

**
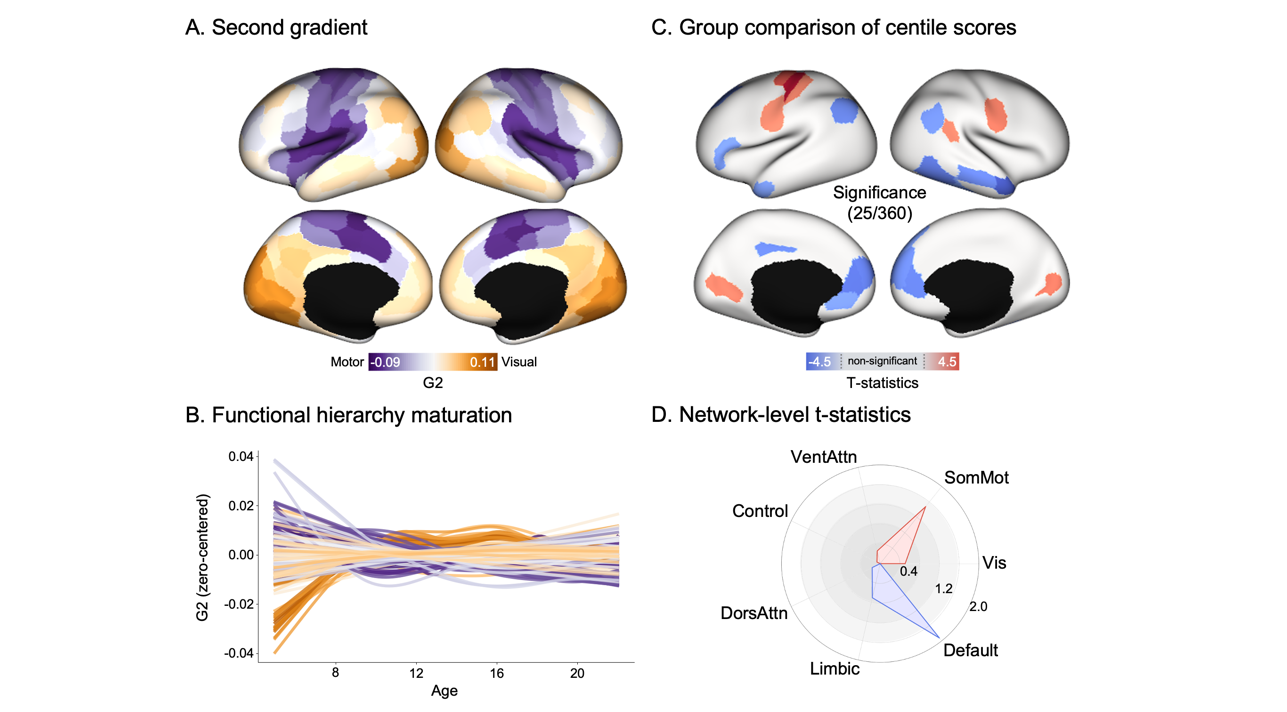
**

**Supplementary Fig. 5 | Atypical development of the cortical hierarchy for the second gradient.** **(A)** The second gradient derived from functional connectivity of the TD group. **(B)** The maturational trajectory of the whole-brain functional hierarchy. Brain regions with significant age effects are plotted ($P_{FDR} < 0.05$), and the color of each line corresponds to the gradient values from (A). **(C)** Whole-brain statistics of group differences in centile scores between ASD and TD groups. **(D)** The t-statistics are stratified using canonical functional networks.
Abbreviations: Vis, Visual; SomMot, Somatomotor; DorsAttn, Dorsal attention; VentAttn, Ventral attention; TD, typically developing; ASD, Autism spectrum disorder.**
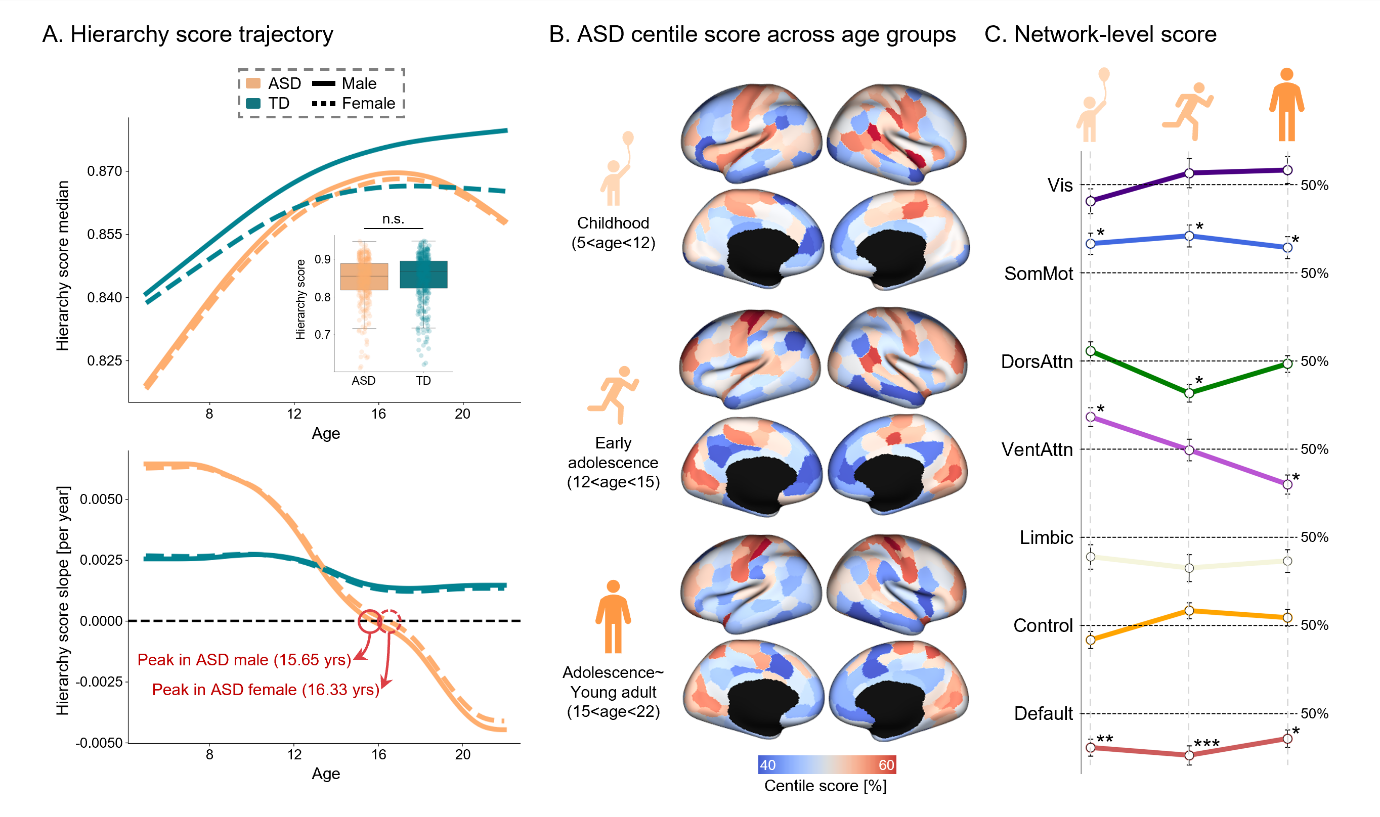
**

**Supplementary Fig. 6 | Hierarchy score analysis derived from the second gradient.** (**A)** Calculation of hierarchy scores and the trajectory of median and slope values [per year]. **(B)** Whole-brain centile scores across three developmental stages. **(C)** Network-level stratification of whole-brain centile scores across different developmental stages. ∗, $p<0.05$; ∗∗, $p<0.01$; ∗∗∗, $p<0.001$.
Abbreviations: TD, typically developing; ASD, Autism spectrum disorder; Vis, Visual; SomMot, Somatomotor; DorsAttn, Dorsal attention; VentAttn, Ventral attention.


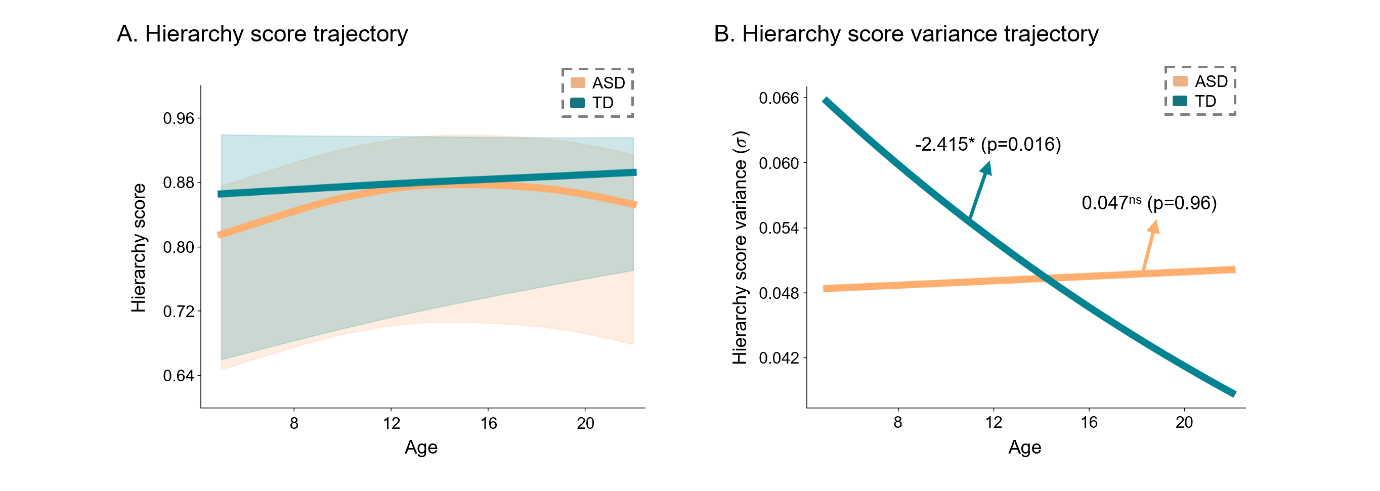


**Supplementary Fig. 7 | The variance in hierarchy score trajectory. (A)** The hierarchy score trajectory with 2.5% and 97.5% centiles. **(B)** The variance of hierarchy score trajectory. ∗, $p<0.05$; ns, not significant.
Abbreviations: TD, typically developing; ASD, Autism spectrum disorder.


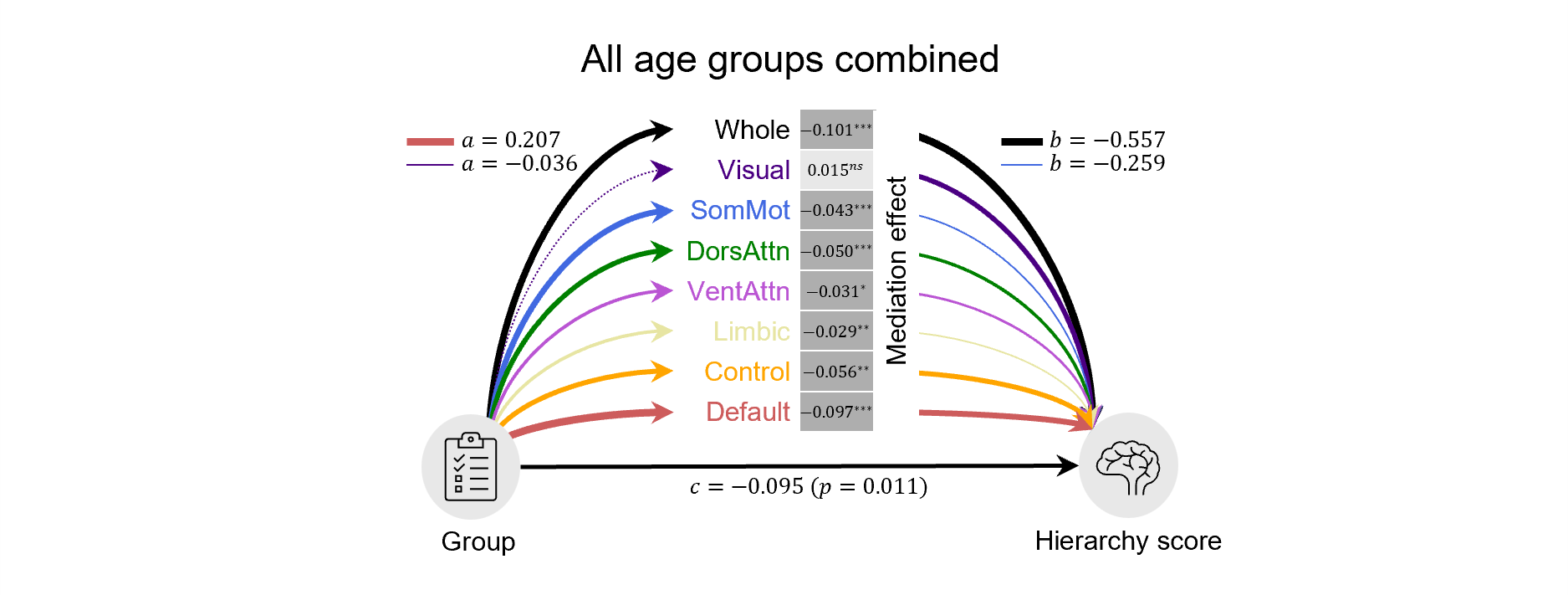


**Supplementary Fig. 8 | Results of mediation analysis in all age groups.** The paths *a, b*, and *c* denote direct effect between two variables. Significant age effects are indicated by darker boxes.
Abbreviations: Whole, whole-brain; Vis, Visual; SomMot, Somatomotor; DorsAttn, Dorsal attention; VentAttn, Ventral attention; Whole, Whole-brain.


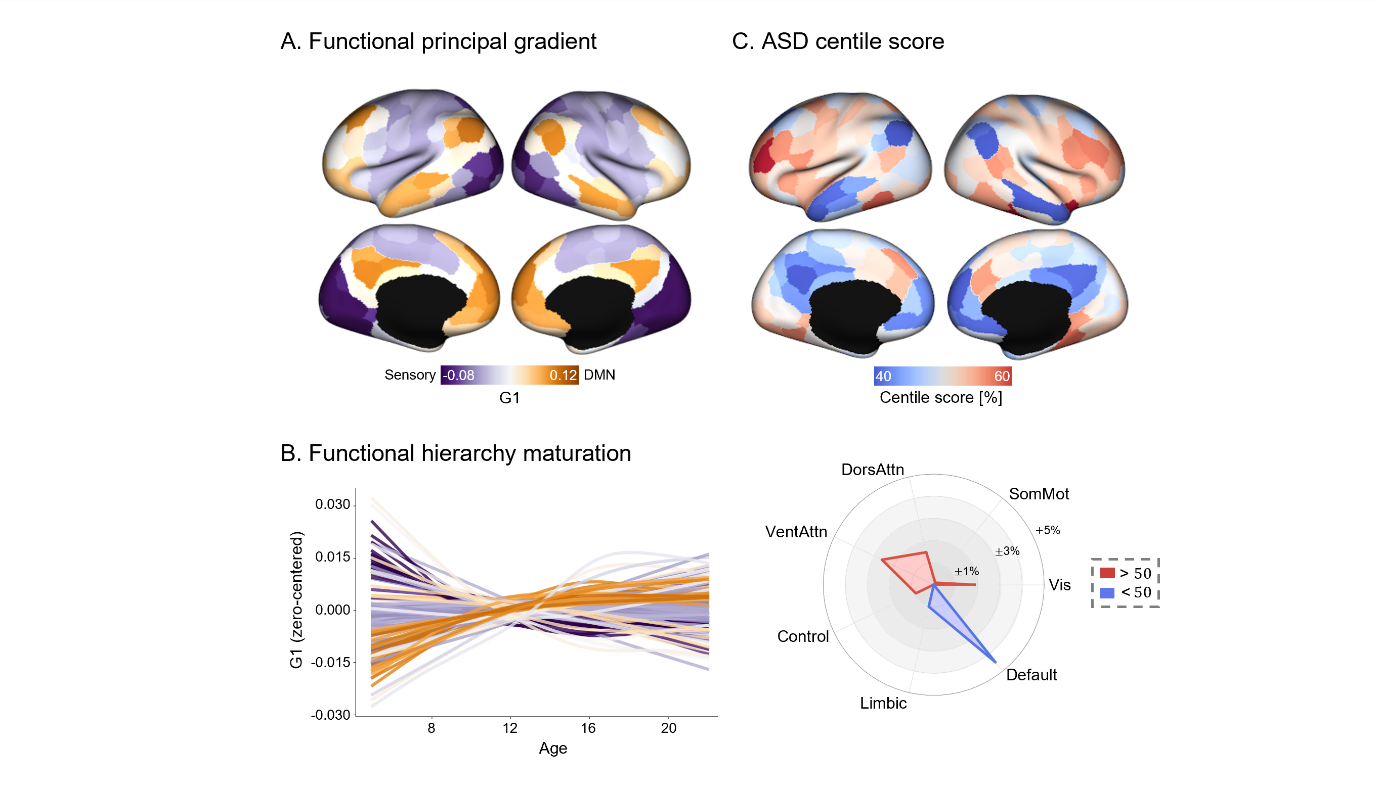


**Supplementary Fig. 9 | Atypical development of the cortical hierarchy in ASD replicated using the ABIDE-II dataset.** **(A)** Principal gradient derived from functional connectivity of the TD group. **(B)** Maturational trajectory of the whole-brain functional hierarchy. Brain regions with significant age effects are plotted ($P_{FDR} < 0.05$), with the color of each line corresponding to the gradient values from (A). **(C)** Centile scores relative to the normative trajectory were calculated to characterize the abnormal development in ASD.
Abbreviations: Vis, Visual; SomMot, Somatomotor; DorsAttn, Dorsal attention; VentAttn, Ventral attention; Whole, Whole-brain; TD, typically developing; ASD, Autism spectrum disorder; ABIDE, Autism Brain Imaging Data Exchange; ROI, region of interest.


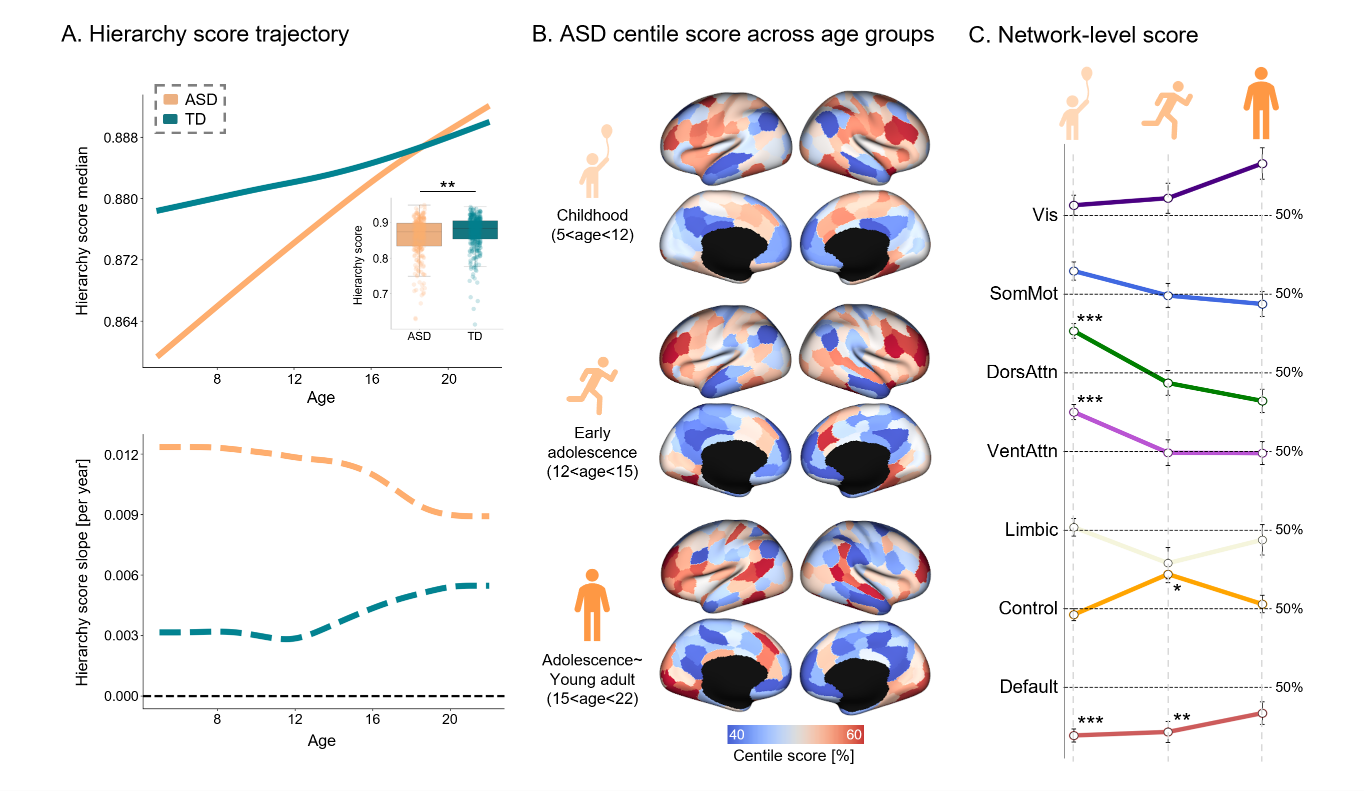


**Supplementary Fig. 10 | Hierarchy score analysis replicated using the ABIDE-II dataset.** **(A)** Calculation of hierarchy scores and the trajectory of median and slope values [per year]. **(B)** Whole-brain centile scores across three developmental stages. **(C)** Network-level stratification of whole-brain centile scores across the stages.
Abbreviations: Vis, Visual; SomMot, Somatomotor; DorsAttn, Dorsal attention; VentAttn, Ventral attention; Whole, Whole-brain; TD, typically developing; ASD, Autism spectrum disorder; ABIDE, Autism Brain Imaging Data Exchange.

**Supplementary Fig. 11 | Hierarchy score analysis in ABIDE-II longitudinal dataset. (A)** Calculation of the hierarchy scores and the trajectory of median at group- and individual levels and **(B)** slop values [per year].
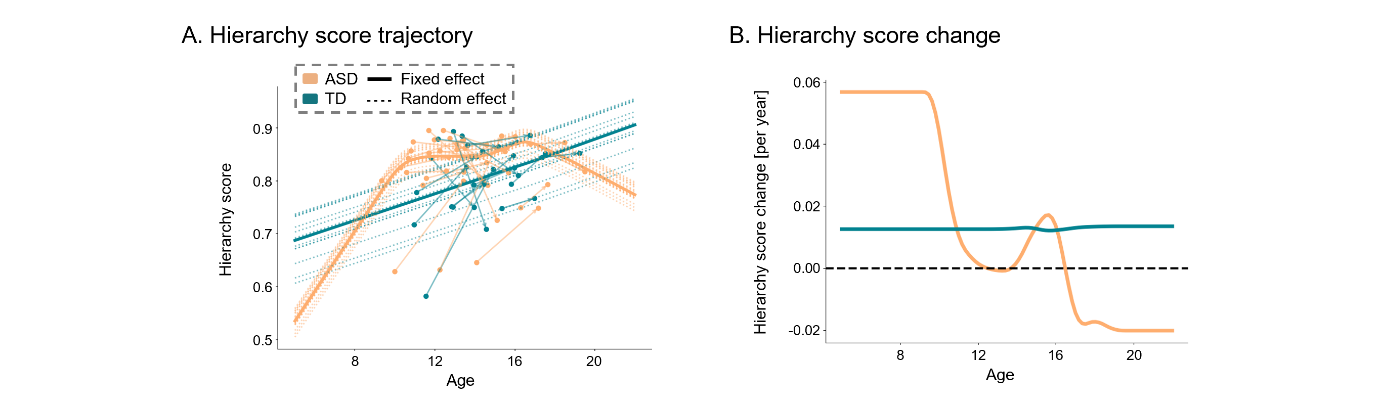


**
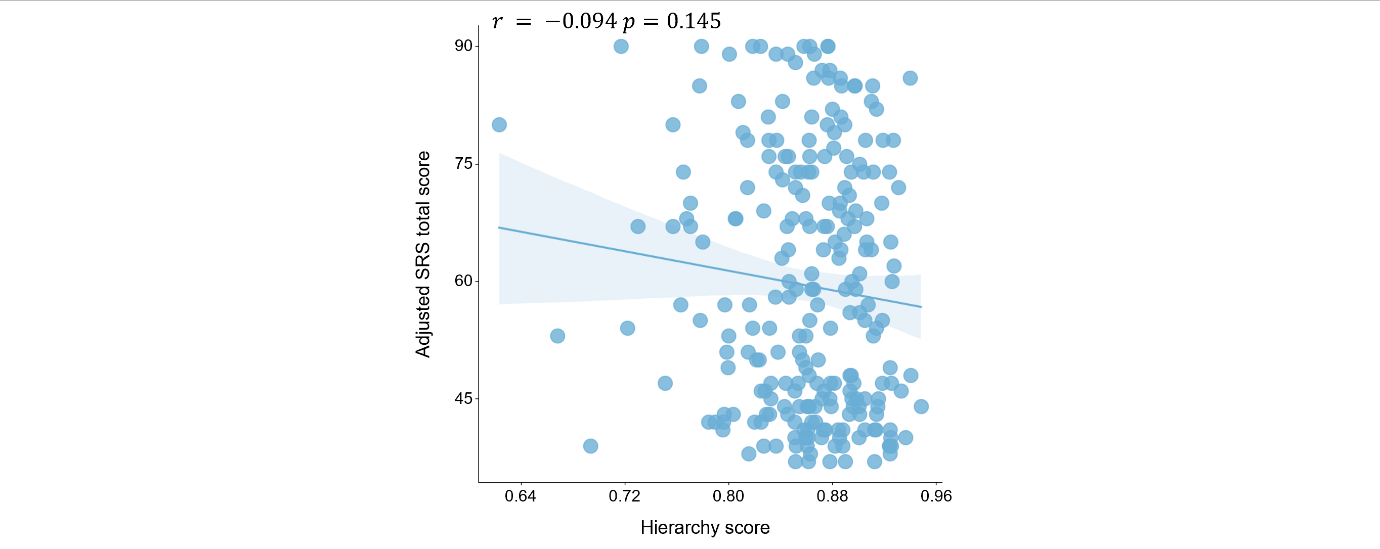
**

**Supplementary Fig. 12 | Correlations between the hierarchy score and symptom severity for ABIDE-I.** Scatter plots between the hierarchy score and total social responsiveness scale (SRS) T-score.


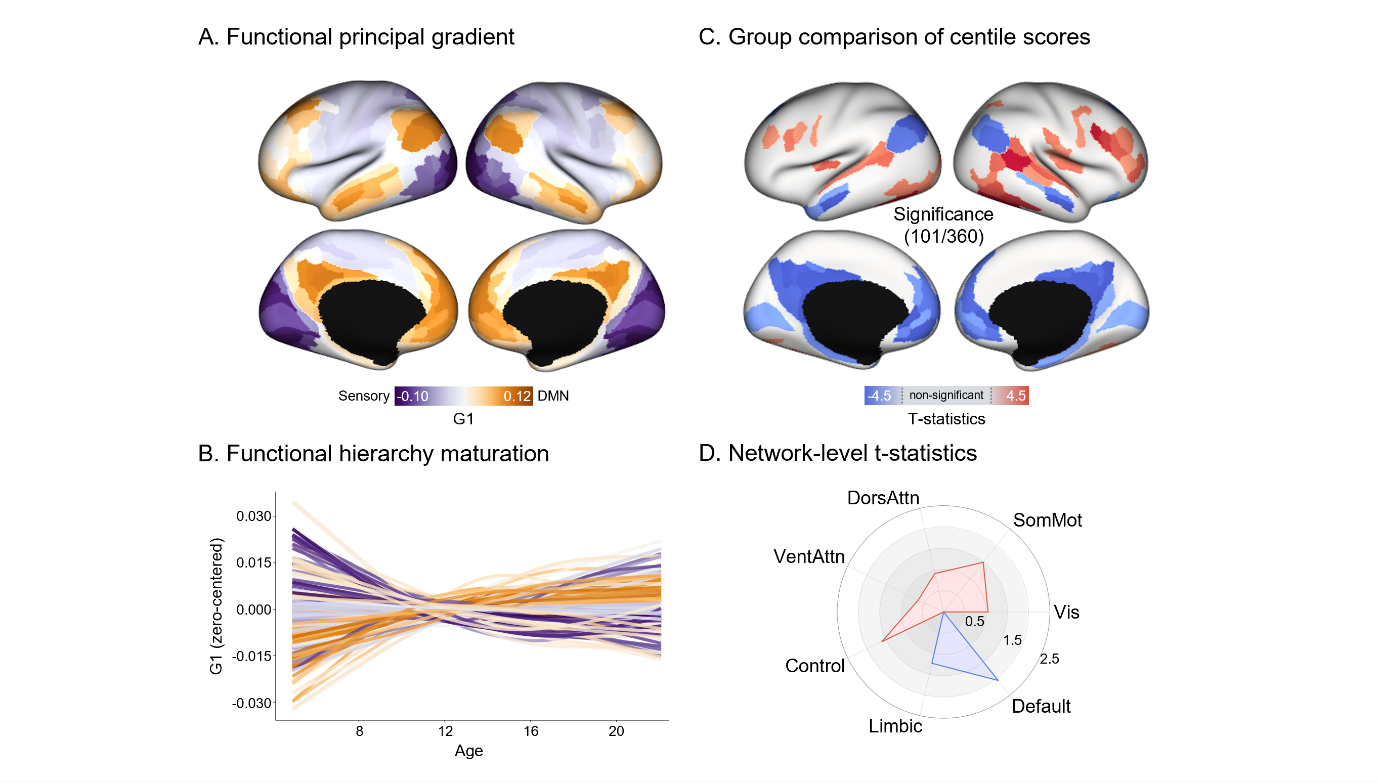


**Supplementary Fig. 13 | Atypical development of the cortical hierarchy replicated using the Glasser atlas.** **(A)** The principal gradient derived from functional connectivity of the TD group. **(B)** The maturational trajectory of the whole-brain functional hierarchy. Brain regions with significant age effects are plotted ($P_{FDR} < 0.05$), and the color of each line corresponds to the gradient values from (A). **(C)** Whole-brain statistics of group differences in centile scores between ASD and TD groups. The number of significant regions (X) is displayed as (X/360). **(D)** The t-statistics are stratified using canonical functional networks.
Abbreviations: Vis, Visual; SomMot, Somatomotor; DorsAttn, Dorsal attention; VentAttn, Ventral attention; TD, typically developing; ASD, Autism spectrum disorder.

**
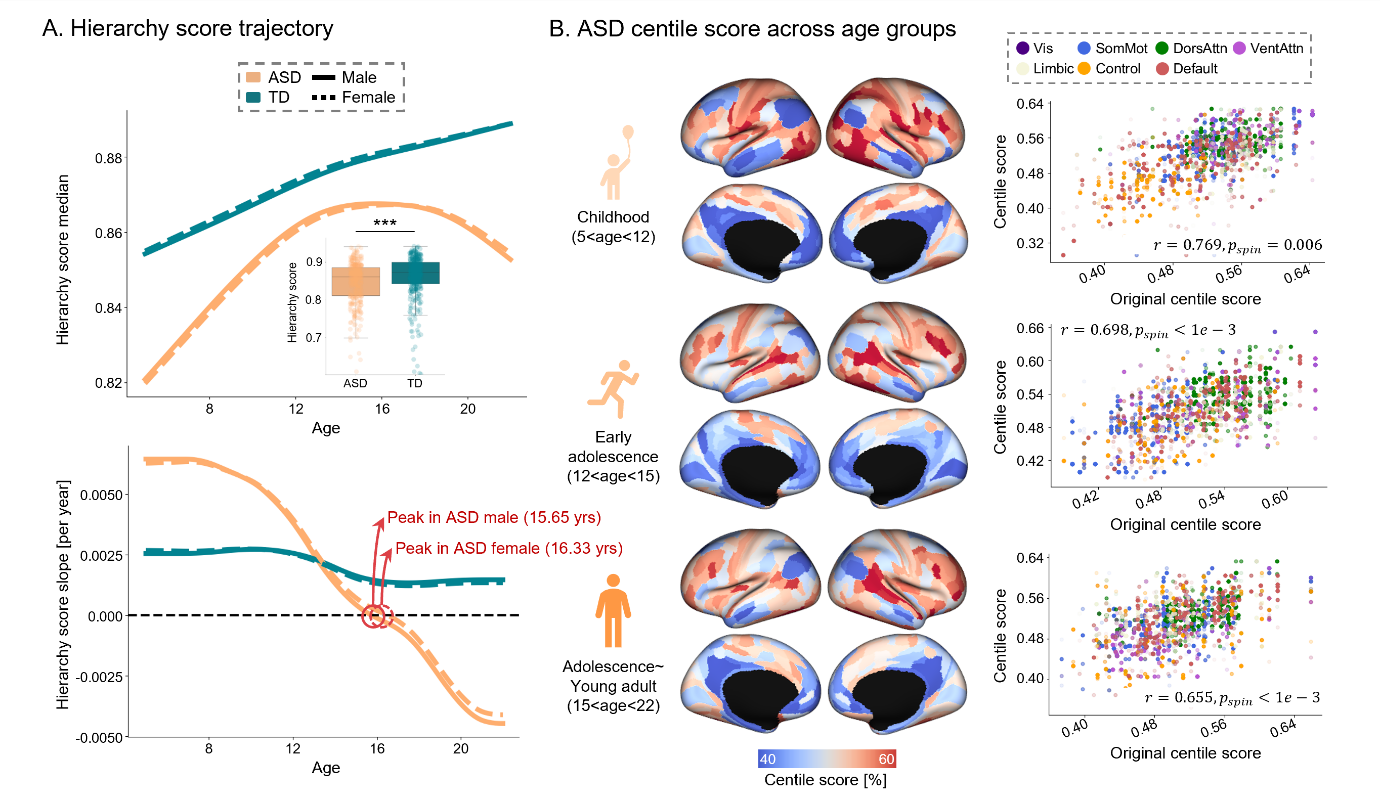
**

**Supplementary Fig. 14 | Hierarchy score analysis using the Glasser atlas. (A)** Calculation of hierarchy scores and the trajectory of median and slop values [per year]. **(B)** Whole-brain centile scores across three developmental stages (*left*) and their spatial correlations between the original model and the model using the Glasser atlas (*right*).

Abbreviations: FD, framewise displacement; TD, typically developing; ASD, Autism spectrum disorder; r, Pearson correlation coefficient.

**
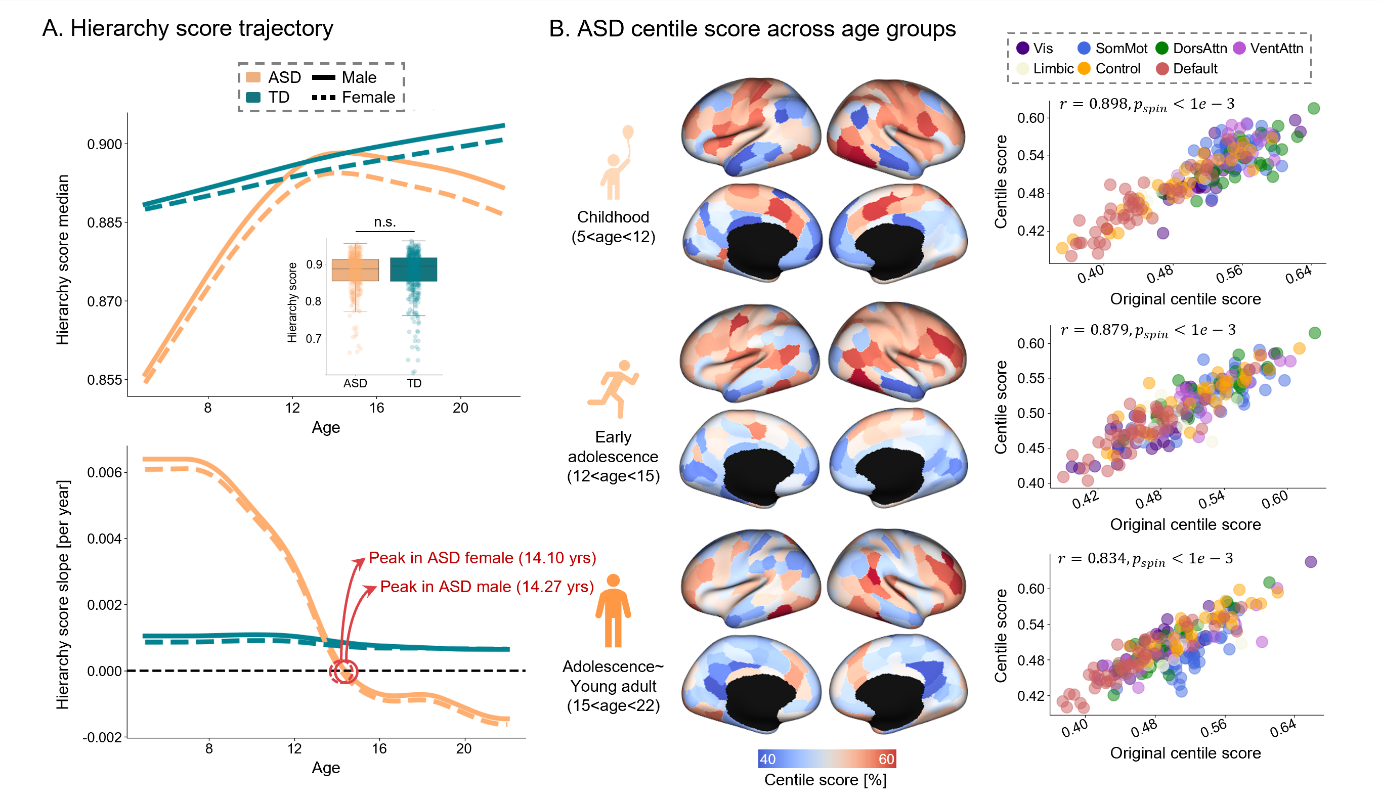
**

**Supplementary Fig. 15 | Hierarchy score analysis using a 5% threshold. (A)** Calculation of hierarchy scores and the trajectory of median and slop values [per year]. **(B)** Whole-brain centile scores across three developmental stages (*left*) and their spatial correlations between the original model and the model based on the connectivity matrices threshold with a 5% threshold (*right*).

Abbreviations: FD, framewise displacement; TD, typically developing; ASD, Autism spectrum disorder; r, Pearson correlation coefficient.

**
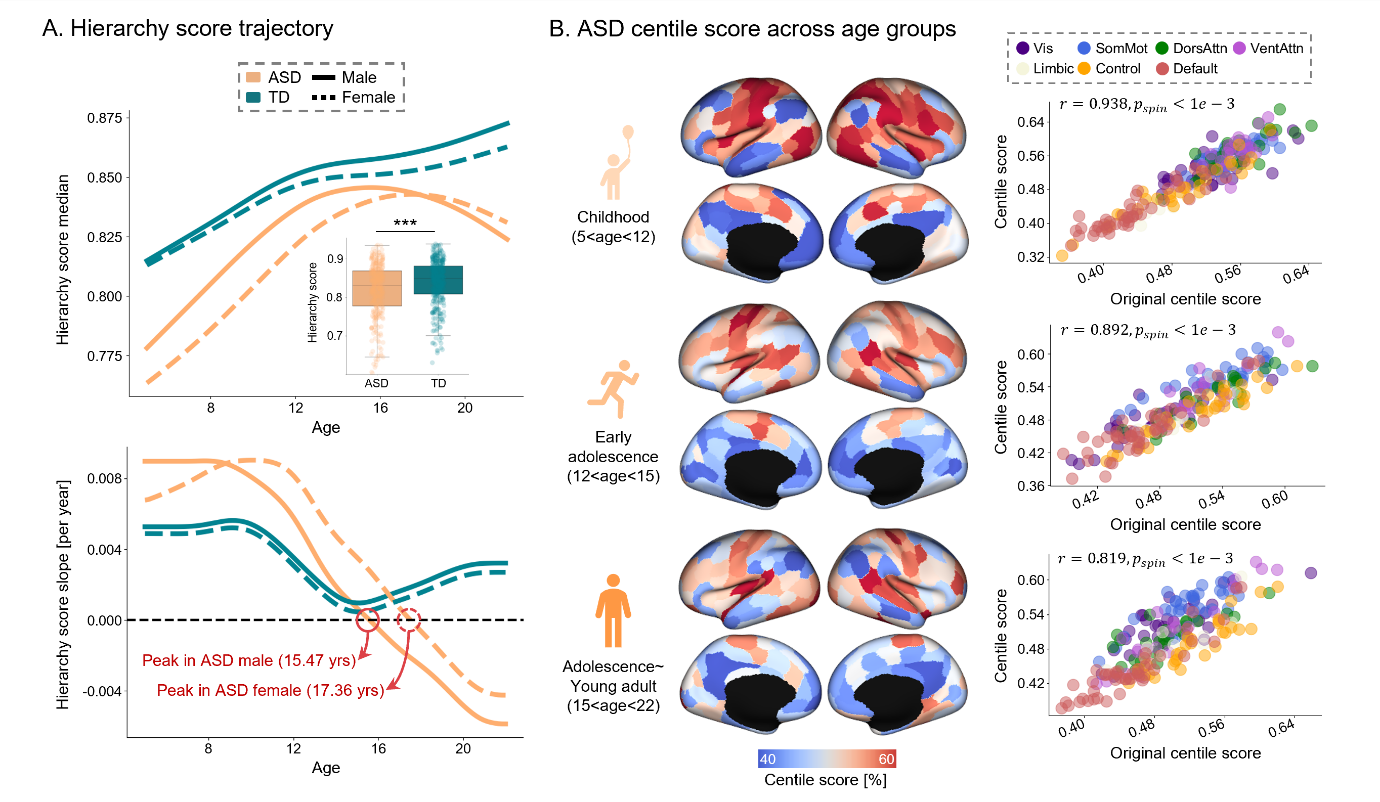
**

**Supplementary Fig. 16 | Hierarchy score analysis using a 20% threshold. (A)** Calculation of hierarchy scores and the trajectory of median and slop values [per year]. **(B)** Whole-brain centile scores across three developmental stages (*left*) and their spatial correlations between the original model and the model based on the connectivity matrices threshold with a 20% threshold (*right*).

Abbreviations: FD, framewise displacement; TD, typically developing; ASD, Autism spectrum disorder; r, Pearson correlation coefficient.


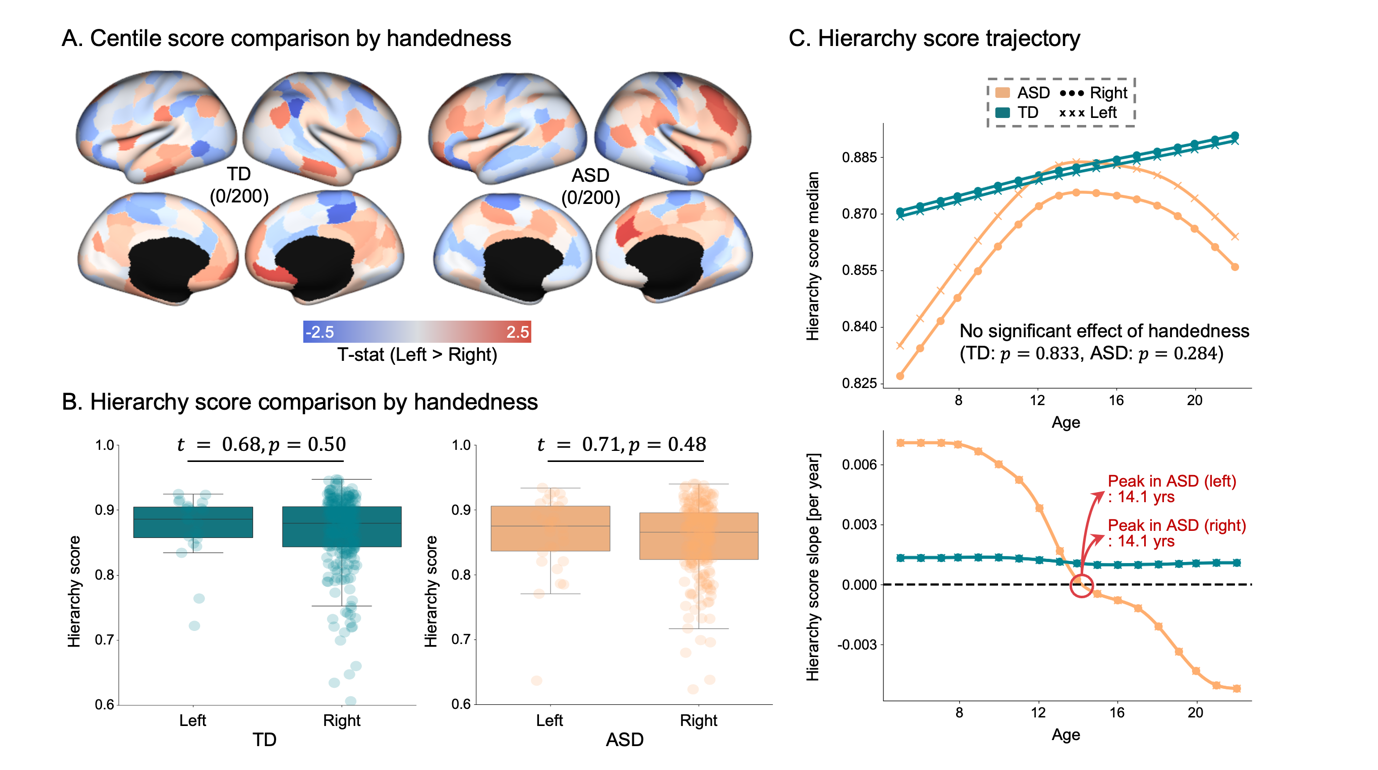


**Supplementary Fig. 17 | Effects of handedness. (A)** Whole-brain statistics of group differences between the left- and right-handed participants using centile scores. The number of significant regions (X) is displayed as (X/200). **(B)** Box plots of hierarchy scores between left- and right-handed participants. **(C)** Calculation of hierarchy scores and the trajectory of median and slope values [per year].
Abbreviations: TD, typically developing; ASD, Autism spectrum disorder.

**
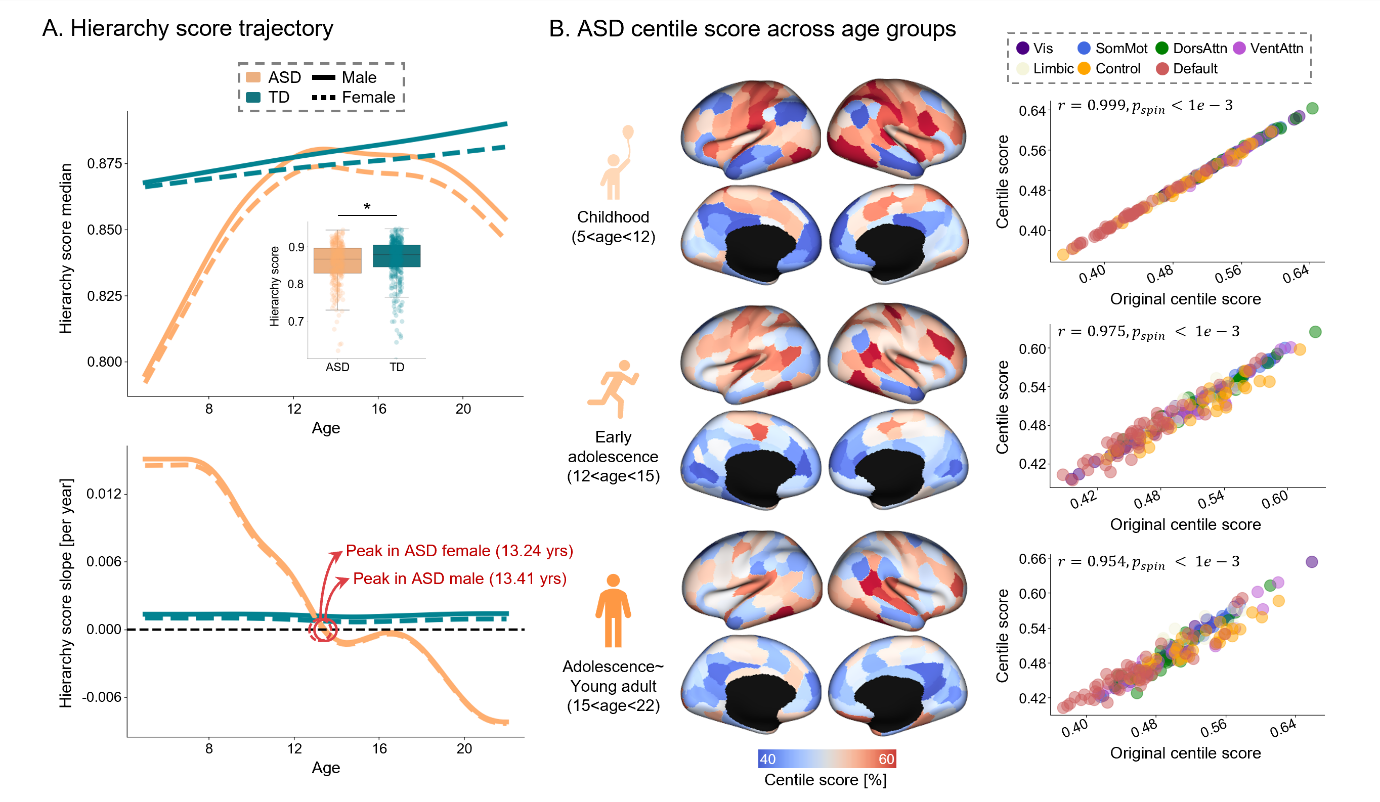
**

**Supplementary Fig. 18 | Hierarchy score analysis after controlling for head motion. (A)** Calculation of hierarchy scores and the trajectory of median and slop values [per year]. **(B)** Whole-brain centile scores across three developmental stages (*left*) and their spatial correlations between the original model and the model controlled for mean FD (*right*).

Abbreviations: FD, framewise displacement; TD, typically developing; ASD, Autism spectrum disorder; r, Pearson correlation coefficient.

Supplementary Table 1 | Demographic information of the ABIDE-I participants.

| Site | n | Age | | *p*-value | Sex (M:F) | | *p*-value | SRS Total (T-score) | | *p*-value | Handedness (L:R:L+R) | | *p*-value |
| --- | --- | --- | --- | --- | --- | --- | --- | --- | --- | --- | --- | --- | --- |
|  |  | ASD | TD |  | ASD | TD |  | ASD | TD |  | ASD | TD |  |
| CALTECH | 13 | 20.03±1.31 | 19.74±1.47 | 0.716 | 4:2 | 4:3 | 1 | - | - | - | 0:4:2 | 0:5:2 | 1 |
| CMU | 8 | 20.25±0.96 | 20.75±0.50 | 0.390 | 3:1 | 3:1 | 1 | - | - | - | 0:4:0 | 0:4:0 | 1 |
| KKI | 44 | 9.92±1.47 | 10.01±1.16 | 0.830 | 13:3 | 20:8 | 0.719 | - | - | - | 1:13:2 | 1:24:3 | 1 |
| LEUVEN_1 | 11 | 19.25±0.89 | 20.00±1.73 | 0.353 | 8:0 | 3:0 | 1 | 64.38±6.90 | 48.00±7.94 | 0.008 | 0:8:0 | 0:3:0 | 1 |
| LEUVEN_2 | 34 | 13.92±1.31 | 14.22±1.45 | 0.535 | 12:3 | 14:5 | 1 | 73.13±13.05 | 44.58±6.40 | <0.001 | 2:13:0 | 3:15:1 | 1 |
| MAX_NUM | 10 | 12.71±4.11 | 9.33±2.08 | 0.223 | 7:0 | 3:0 | 1 | - | - | - | 0:7:0 | 0:3:0 | 1 |
| NYU | 135 | 11.98±3.35 | 13.21±3.76 | 0.050 | 53:5 | 59:18 | 0.036 | 72.23±11.50 | 45.41±5.33 | <0.001 | - | - | - |
| OHSU | 24 | 11.43±2.18 | 10.24±1.13 | 0.109 | 12:0 | 12:0 | 1 | - | - | - | 1:11:0 | 0:12:0 | 1 |
| OLIN | 25 | 16.64±2.82 | 16.36±3.35 | 0.832 | 12:2 | 9:2 | 1 | - | - | - | 3:11:0 | 1:10:0 | 0.598 |
| PITT | 38 | 15.39±3.66 | 15.92±3.06 | 0.632 | 16:3 | 15:4 | 1 | - | - | - | 2:17:0 | 1:18:0 | 1 |
| SDSU | 32 | 15.25±1.52 | 14.22±1.90 | 0.144 | 10:0 | 16:6 | 0.142 | - | - | - | 1:9:0 | 3:19:0 | 1 |
| STANFORD | 35 | 10.29±1.60 | 9.89±1.62 | 0.470 | 12:4 | 15:4 | 1 | - | - | - | 3:12:1 | 0:17:2 | 0.183 |
| TRINITY | 38 | 16.57±2.88 | 16.42±2.94 | 0.875 | 19:0 | 19:0 | 1 | - | - | - | 0:19:0 | 0:19:0 | 1 |
| UCLA_1 | 59 | 13.49±2.61 | 13.33±2.11 | 0.800 | 28:4 | 23:4 | 1 | - | - | - | 3:29:0 | 3:24:0 | 1 |
| UCLA_2 | 19 | 12.81±2.02 | 12.33±1.05 | 0.505 | 8:0 | 9:2 | 0.485 | - | - | - | 2:6:0 | 1:10:0 | 0.546 |
| UM_1 | 76 | 13.57±2.50 | 14.05±3.15 | 0.483 | 24:8 | 29:15 | 0.455 | - | - | - | 6:22:1 | 6:35:0 | 0.415 |
| UM_2 | 26 | 14.85±1.62 | 15.54±1.66 | 0.295 | 11:1 | 13:1 | 1 | - | - | - | 1:10:0 | 1:13:0 | 1 |
| USM | 30 | 17.56±2.59 | 16.65±2.03 | 0.379 | 22:0 | 8:0 | 1 | 75.41±8.79 | 43.00±5.07 | <0.001 | - | - |  |
| YALE | 45 | 12.91±3.00 | 12.75±2.99 | 0.859 | 17:6 | 17:5 | 1 | 74.38±9.67 | 46.76±88.97 | <0.001 | 5:18:0 | 3:19:0 | 0.700 |
| **Total** | 702 | 13.91±3.58 | 13.66±3.49 | 0.353 | 291:42 | 291:78 | 0.004 | 72.76±10.87 | 45.42±6.31 | <0.001 | 30:213:6 | 23:250:8 | 0.312 |

The *p*-values for age were derived from a two-sample t-test, and those for sex were derived from Fisher’s exact test. Ambidextrous participants were excluded.
Abbreviations: TD, typically developing; ASD, Autism spectrum disorder; ABIDE, Autism Brain Imaging Data Exchange; M, male; F, female; SRS, social responsiveness scale; L, left; R, right.

**Supplementary Table 2 |** **Demographic information of the ABIDE-II participants.**

| Site | n | Age | | *p*-value | Sex (M:F) | | *p*-value | SRS Total (T-score) | | *p*-value | Handedness (L:R:L+R) | | *p*-value |
| --- | --- | --- | --- | --- | --- | --- | --- | --- | --- | --- | --- | --- | --- |
|  |  | ASD | TD |  | ASD | TD |  | ASD | TD |  | ASD | TD |  |
| BNI | 14 | 19.38±1.19 | 19.33±1.37 | 0.952 | 8:0 | 6:0 | 1 | 71.00±12.31 | 54.83±16.82 | 0.094 | 0:8:0 | 0:6:0 | 1 |
| EMC | 28 | 8.59±1.22 | 8.01±1.06 | 0.191 | 12:3 | 11:2 | 1 | - | - | - | 3:12:0 | 4:9:0 | 0.670 |
| GU | 56 | 11.11±1.72 | 10.53±1.81 | 0.226 | 23:2 | 16:15 | 0.001 | 73.56±15.60 | 44.35±7.03 | <0.001 | 3:22:0 | 1:30:0 | 0.314 |
| IP | 24 | 14.95±4.14 | 15.88±5.11 | 0.629 | 9:5 | 1:9 | 0.013 | - | - | - | 0:14:0 | 2:8:0 | 0.163 |
| IU | 15 | 18.75±0.89 | 20.29±0.76 | 0.003 | 8:0 | 5:2 | 0.200 | - | - | - | 0:8:0 | 0:7:0 | 1 |
| KKI | 184 | 10.58±1.48 | 10.43±1.18 | 0.461 | 32:11 | 87:54 | 0.147 | 76.07±10.42 | 43.28±5.52 | <0.001 | 2:38:3 | 7:124:10 | 1 |
| KUL_3 | 10 | 19.20±1.23 | - | - | 10:0 | - | - | 58.20±9.59 | - | - | 3:7:0 | - | - |
| NYU_1 | 72 | 8.80±2.81 | 9.03±1.98 | 0.706 | 40:3 | 28:1 | 0.644 | 75.74±16.51 | 45.32±6.12 | <0.001 | 2:24:7 | 0:27:0 | 0.005 |
| NYU_2 | 24 | 6.61±0.98 | - | - | 22:2 | - | - | 78.63±14.26 | - | - | 4:11:7 | - |  |
| OHSU | 87 | 11.80±2.32 | 10.38±1.67 | 0.001 | 28:7 | 28:24 | 0.014 | 75.74±11.57 | 46.52±7.12 | <0.001 | 1:33:1 | 0:52:0 | 0.159 |
| SDSU | 46 | 13.08±3.20 | 13.56±3.05 | 0.615 | 22:5 | 17:2 | 0.682 | 83.12±9.06 | 43.53±5.15 | <0.001 | 3:23:1 | 0:17:2 | 0.285 |
| TCD | 32 | 14.95±3.36 | 16.13±2.81 | 0.287 | 15:0 | 17:0 | 1 | 77.53±8.59 | 41.88±6.06 | <0.001 | 0:15:0 | 0:17:0 | 1 |
| UCD | 28 | 15.14±1.87 | 15.00±1.61 | 0.836 | 12:3 | 9:4 | 0.670 | 70.67±11.92 | 41.00±2.57 | <0.001 | 0:14:1 | 0:13:0 | 1 |
| UCLA | 25 | 12.46±1.79 | 9.61±1.97 | 0.001 | 12:0 | 9:4 | 0.096 | - | - | - | 1:11:0 | 1:11:1 | 1 |
| USM | 12 | 16.30±2.54 | 15.54±3.59 | 0.680 | 7:1 | 4:0 | 1 | 83.00±8.77 | 46.00±7.12 | 0.001 | 0:5:2 | 0:3:0 | 1 |
| **Total** | 657 | 11.93±4.01 | 11.36±3.20 | 0.043 | 260:42 | 234:121 | <0.001 | 75.77±13.58 | 44.17±6.71 | <0.001 | 22:245:22 | 15:324:14 | 0.020 |

The *p*-values for age and SRS score were derived from a two-sample t-test, and those for sex and handedness were derived from Fisher’s exact test. Abbreviations: TD, typically developing; ASD, Autism spectrum disorder; ABIDE, Autism Brain Imaging Data Exchange; SRS, social responsiveness scale; M, male; F, female; SRS, social responsiveness scale; L, left; R, right.

**Supplementary Table 3 |** **Demographic information of the ABIDE-II longitudinal participants.**

| Stie | n | Follow-up year | Age at baseline | | *p*-value | Sex (M:F) | | *p*-value | Handedness (L:R:LR) | | *p*-value |
| --- | --- | --- | --- | --- | --- | --- | --- | --- | --- | --- | --- |
|  |  |  | ASD | TD |  | ASD | TD |  | ASD | TD |  |
| UCLA | 21 | 2.89±0.34 | 11.73±1.19 | 12.23±1.09 | 0.363 | 13:1 | 7:0 | 1 | 1:13:0 | 0:7:0 | 1 |
| UPSM | 17 | 1.75±0.20 | 13.74±2.74 | 14.30±1.82 | 0.630 | 7:2 | 6:2 | 1 | 1:7:1 | 1:7:0 | 1 |
| **Total** | 38 | 2.38±0.63 | 12.52±2.14 | 13.34±1.82 | 0.230 | 20:3 | 13:2 | 1 | 2:20:1 | 1:14:0 | 1 |

The *p*-values for age were derived from a two-sample t-test, and those for sex and handedness were derived from Fisher’s exact test.
Abbreviations: TD, typically developing; ASD, Autism spectrum disorder; ABIDE, Autism Brain Imaging Data Exchange; M, male; F, female; L, left; R, right.

**Supplementary Table 4 | Site-specific imaging parameters of the ABIDE-I participants.**

| Site | n | T1-weighted | | | | | | Resting-state fMRI | | | | | |
| --- | --- | --- | --- | --- | --- | --- | --- | --- | --- | --- | --- | --- | --- |
|  |  | TR (ms) | TE (ms) | FA (deg) | FoV (mm^2^) | Res (mm^3^) | TR (ms) | | TE (ms) | FA (deg) | FoV (mm^2^) | Res (mm^3^) |  |
| CALTECH | 13 | 1590 | 2.73 | 10 | 256$\times$256 | 1$\times$1$\times$1 | 2000 | | 30 | 75 | 224$\times$224 | 3.5$\times$3.5$\times$3.5 |  |
| CMU | 8 | 1870 | 2.48 | 8 | 256$\times$256 | 1$\times$1$\times$1 | 2000 | | 30 | 73 | 192$\times$192 | 3$\times$3$\times$3 |  |
| KKI | 44 | 3500 | 3.7 | 8 | 256$\times$200 | 1$\times$1$\times$1 | 2500 | | 30 | 75 | 256$\times$256 | 3$\times$3$\times$3 |  |
| LEUVEN_1 | 11 | 3000 | 4.6 | 8 | 250$\times$250 | 0.98$\times$0.98$\times$0.98 | 1667 | | 33 | 90 | 230$\times$230 | 3.59$\times$3.59$\times4$ |  |
| LEUVEN_2 | 34 | 3000 | 4.6 | 8 | 250$\times$250 | 0.98$\times$0.98$\times$0.98 | 1667 | | 33 | 90 | 230$\times$230 | 3.59$\times$3.59$\times4$ |  |
| MAX_NUM | 10 | 1800 | 3.06 | 9 | 256$\times$240 | 1$\times$1$\times$1 | 3000 | | 30 | 80 | 192$\times$192 | 3$\times$3$\times$4 |  |
| NYU | 135 | 2530 | 3.25 | 7 | 256$\times$192 | 1.3$\times$1$\times$1.3 | 2000 | | 15 | 90 | 240$\times$192 | 3$\times$3$\times$4 |  |
| OHSU | 24 | 2300 | 3.59 | 10 | 256$\times$240 | 1$\times$1$\times$1.1 | 2500 | | 30 | 90 | 240$\times$240 | 3.8$\times$3.8$\times$3.8 |  |
| OLIN | 25 | 2500 | 2.74 | 8 | 256$\times$208 | 1$\times$1$\times$1 | 1500 | | 27 | 60 | 220$\times$220 | 3.4$\times$3.4$\times$3.4 |  |
| PITT | 38 | 2100 | 3.93 | 7 | 256$\times$256 | 1.1$\times$1.1$\times$1.1 | 1500 | | 25 | 70 | 200$\times$200 | 3.1$\times$3.1$\times$3.4 |  |
| SDSU | 32 | 11.08 | 4.3 | 8 | 256$\times$256 | 1$\times$1$\times$1 | 2000 | | 30 | 90 | 220$\times$220 | 3.4$\times$3.4$\times$3.4 |  |
| STANFORD | 35 | 8.4 | 1.8 | 15 | 220$\times$220 | 0.859$\times$1.5$\times$0.859 | 2000 | | 30 | 80 | 200$\times$200 | 3.125$\times$3.125$\times$4.5 |  |
| TRINITY | 38 | 3000 | 3.9 | 8 | 256$\times$256 | 1$\times$1$\times$1 | 2000 | | 28 | 90 | 240$\times$240 | 3$\times$3$\times$3.5 |  |
| UCLA_1 | 59 | 2300 | 2.84 | 9 | 256$\times$240 | 1$\times$1$\times$1.2 | 3000 | | 28 | 90 | 192$\times$192 | 3$\times$3$\times$4 |  |
| UCLA_2 | 19 | 2300 | 2.84 | 9 | 256$\times$240 | 1$\times$1$\times$1.2 | 3000 | | 28 | 90 | 192$\times$192 | 3$\times$3$\times$4 |  |
| UM_1 | 76 | - | 1.8 | 15 | 256$\times$256 | 1.02$\times$1.02$\times$1.4 | 2000 | | 30 | 90 | 220$\times$220 | 3.438$\times$3.438$\times$3 |  |
| UM_2 | 26 | - | 1.8 | 15 | 256$\times$256 | 1.02$\times$1.02$\times$1.4 | 2000 | | 30 | 90 | 220$\times$220 | 3.438$\times$3.438$\times$3 |  |
| USM | 30 | 2300 | 2.91 | 9 | 256$\times$240 | 1$\times$1$\times$1.2 | 2000 | | 28 | 90 | 220$\times$220 | 3.4$\times$3.4$\times$3.3 |  |
| YALE | 45 | 1230 | 1.73 | 9 | 250$\times$250 | 1$\times$1$\times$1 | 2000 | | 25 | 60 | 220$\times$220 | 3.4$\times$3.4$\times$3.4 |  |

Abbreviations: TR, repetition time; TE, echo time; FA, flip angle; FoV, field of view; Res, resolution; ABIDE, Autism Brain Imaging Data Exchange; fMRI, functional magnetic resonance imaging.

**Supplementary Table 5 | Site-specific imaging parameters of the ABIDE-II participants.**

| Site | n | T1-weighted | | | | | | Resting-state fMRI | | | | | |
| --- | --- | --- | --- | --- | --- | --- | --- | --- | --- | --- | --- | --- | --- |
|  |  | TR (ms) | TE (ms) | FA (deg) | FoV (mm^2^) | Res (mm^3^) | TR (ms) | | TE (ms) | FA (deg) | FoV (mm^2^) | Res (mm^3^) |  |
| BNI | 14 | 6.7 | 3.1 | 9 | 270$\times$252 | 1.1$\times$1.1$\times$1.2 | 3000 | | 25 | 80 | 240$\times$240 | 3.75$\times$3.75$\times$3.75 |  |
| EMC | 28 | 10.26 | 4.236 | 16 | 230$\times$230 | 0.9$\times$0.9$\times$0.9 | 2000 | | 30 | 85 | 230$\times$230 | 3.59$\times$3.59$\times$3.59 |  |
| GU | 56 | 2530 | 3.5 | 7 | 256$\times$256 | 1$\times$1$\times$1 | 2000 | | 30 | 90 | 192$\times$192 | 3$\times$3$\times$2.5 |  |
| IP | 24 | 25 | 5.6 | 30 | 240$\times$240 | 1$\times$1$\times$1 | 2700 | | 45 | 90 | 230$\times$230 | 3.59$\times$3.65$\times$4 |  |
| IU | 15 | 2400 | 2.3 | 8 | 224$\times$224 | 0.7$\times$0.7$\times$0.7 | 813 | | 28 | 60 | 220$\times$220 | 3.4$\times$3.4$\times$3.4 |  |
| KKI | 184 | 3500 | 3.5 | 8 | 256$\times$200 | 1$\times$1$\times$1 | 2500 | | 30 | 75 | 256$\times$256 | 3$\times$3$\times$3 |  |
| KUL_3 | 10 | 9.4 | 4.6 | 8 | 250$\times$250 | 1.2$\times$1.2$\times$1.2 | 2500 | | 30 | 90 | 200$\times$200 | 2.5$\times$2.56$\times$1.2.7 |  |
| NYU_1 | 72 | 3.25 | 3.25 | 7 | 256$\times$256 | 1.3$\times$1$\times$1.33 | 2000 | | 30 | 82 | 240$\times$240 | 3$\times$3$\times$4 |  |
| NYU_2 | 24 | 3.25 | 3.25 | 7 | 256$\times$256 | 1.3$\times$1$\times$1.33 | 2000 | | 15 | 90 | 240$\times$240 | 3$\times$3$\times$3 |  |
| OHSU | 87 | 2300 | 3.58 | 10 | 256$\times$240 | 1$\times$1$\times$1.1 | 2500 | | 30 | 90 | 240$\times$240 | 3.8$\times$3.8$\times$3.8 |  |
| SDSU | 46 | 8.136 | 3.172 | 8 | 256$\times$256 | 1$\times$1$\times$1 | 2000 | | 30 | 90 | 220$\times$220 | 3.44$\times$3.44$\times$3.4 |  |
| TCD | 32 | 8.4 | 3.9 | 8 | 230$\times$230 | 0.9$\times$0.9$\times$0.9 | 2000 | | 27 | 90 | 240$\times$240 | 3$\times$3$\times$3.2 |  |
| UCD | 28 | 2000 | 3.16 | 8 | 256$\times$224 | 1$\times$1$\times$1 | 2000 | | 24 | 90 | 224$\times$224 | 3.5$\times$3.5$\times$4 |  |
| UCLA | 25 | 2300 | 2.3 | 9 | 256$\times$240 | 1$\times$1$\times$1.2 | 3000 | | 28 | 90 | 192$\times$192 | 3$\times$3$\times$4 |  |
| USM | 12 | 900 | 2.91 | 9 | 256$\times$240 | 1$\times$1$\times$1.2 | 2000 | | 29 | 90 | 220$\times$220 | 3.4$\times$3.4$\times$3 |  |

Abbreviations: TR, repetition time; TE, echo time; FA, flip angle; FoV, field of view; Res, resolution; ABIDE, Autism Brain Imaging Data Exchange; fMRI, functional magnetic resonance imaging.
